# Structures of the free and inhibitors-bound forms of bromelain and ananain from *Ananas comosus* stem and in vitro study of their cytotoxicity

**DOI:** 10.1038/s41598-020-76172-5

**Published:** 2020-11-11

**Authors:** Mohamed Azarkan, Erik Maquoi, François Delbrassine, Raphael Herman, Nasiha M’Rabet, Rafaèle Calvo Esposito, Paulette Charlier, Frédéric Kerff

**Affiliations:** 1 Laboratoire de Chimie Générale (Unité de Chimie Des Protéines), Faculté de Médecine, Université Libre de Bruxelles, Campus Erasme (CP 609), 1070 Bruxelles, Belgium; 2 Laboratoire de Biologie Des Tumeurs Et du Développement, GIGA-Cancer, Université de Liège, 4000 Liège, Belgium; 3 UR InBioS, Centre D’Ingénierie Des Protéines, Université de Liège, Sart Tilman, 4000 Liège, Belgium

**Keywords:** Neuroscience, Diseases of the nervous system, Addiction

## Abstract

The *Ananas* *comosus* stem extract is a complex mixture containing various cysteine ​​proteases of the C1A subfamily, such as bromelain and ananain. This mixture used for centuries in Chinese medicine, has several potential therapeutic applications as anti-cancer, anti-inflammatory and ecchymosis degradation agent. In the present work we determined the structures of bromelain and ananain, both in their free forms and in complex with the inhibitors E64 and TLCK. These structures combined with protease-substrate complexes modeling clearly identified the Glu68 as responsible for the high discrimination of bromelain in favor of substrates with positively charged residues at P2, and unveil the reasons for its weak inhibition by cystatins and E64. Our results with purified and fully active bromelain, ananain and papain show a strong reduction of cell proliferation with MDA-MB231 and A2058 cancer cell lines at a concentration of about 1 μM, control experiments clearly emphasizing the need for proteolytic activity. In contrast, while bromelain and ananain had a strong effect on the proliferation of the OCI-LY19 and HL-60 non-adherent cell lines, papain, the archetypal member of the C1A subfamily, had none. This indicates that, in this case, sequence/structure identity beyond the active site of bromelain and ananain is more important than substrate specificity.

## Introduction

The *Ananas* *comosus* stem extract (often improperly described as stem bromelain) is a complex extract containing various cysteine proteases (iso)forms of the papain family (CA clan, C1 family) and other partially characterized non-proteolytic compounds^1–7^. One of the key challenges faced by researchers studying cysteine proteases, particularly those of plant origin, was the characterization of multiple enzyme (iso)forms, such as those found in *A. comosus* stem extract^8,9^. These multiple proteases, despite having high homology in their primary sequences, show differences in substrate specificity and inhibitory properties. It is therefore interesting to identify the structural modifications that may be linked to such deviations. Cysteine proteases in particular can easily be irreversibly oxidized, e.g. by air, making their separation from active forms very challenging. The preparation of fully active enzymes from mixtures containing inactivated material has been enabled by the use of affinity chromatography^10,11^. However, affinity chromatography is not convenient for both practical and economic reasons for the production of the large quantities of pure proteases required for biophysical, mechanistic and structural investigations. Three different cysteine proteases of the C1A family were usually identified in *A. comosus* stem extracts: basic stem bromelain (the major component), ananain and comosain^2,12,13^. We have recently purified to homogeneity and characterized several catalytically competent species from *A. comosus* stem extracts by using an efficient strategy based on the covalent grafting of an activated polyethylene glycol chain followed by purification on classical chromatographic gel media. This allowed a further separation of the extract into two acidic bromelains, three basic bromelains, two ananains and comosain^14^. Basic bromelains represent the most abundant cysteine proteases fraction of the crude *A. comosus* stem extract^1–7^. Interestingly, basic bromelains are scarcely inhibited by chicken cystatin and slowly inactivated by E64, unlike most cysteine proteinases of the papain family. A number of deletions and mutations have been proposed on the basis of sequence alignment, to explain such uncommon behavior when compared to the archetypal protease, papain^1,15,16^. Comparatively, it has been shown that papain has a much better reactivity for iodoacetate than for iodoacetamide, in particular because imidazolium group of the active thiolate-imidazolium catalytic dyad interacts favorably with the negative charge of the carboxylate group of the alkylating agent. In the case of basic bromelains, the difference in reactivity towards these two compounds is very small compared to papain. These data clearly show that, on the one hand, basic bromelains have a low reactivity towards these alkylating agents and, on the other hand, low discrimination in favor of negatively charged alkylating agents. The fact that basic bromelains are only barely affected by cystatins was attributed to the modification of the structural organization of the catalytic site^16^. However, this interpretation remains elusive in the absence of structural data. In contrast, ananain is distinguished from basic bromelains by both its catalytic specificity and its very high reactivity towards E64^3^.

Therefore, a detailed comparative structural study of ananain, which behaves typically as the archetypal enzyme papain, and the basic bromelains may help understand the described dissimilarities at a molecular level.

Many studies have been conducted with *A. comosus* stem extracts, identifying a wide variety of biological systems affected^17–20^. From these, the anticancer activities are perhaps the most attractive properties, deserving further studies^21–26^. The complexity of *A. comosus* stem extracts does however not allow linking the observed effect to a particular constituent, consequently preventing a precise interpretation and understanding of the molecular mechanisms taking place. A recent study investigated the possible analgesic action of *A. comosus* stem extract by degrading the proenkephalin both, in vitro and in vivo, giving rise to multiple opioid peptides^20^. The generated bio-active peptides were suggested to act in periphery where they can have an anti-inflammatory activity that has been recognized for many decades^20^. This specificity of *A. comosus* stem extract to cleave proenkephalin mimics that of the nervous system prohormones convertases 1 and 2 which specifically and exclusively cleave proenkephalin after basic amino acids pairs^27^. Assays of combinatorial peptide library^28^ and of solution-phase fluorogenic peptide microarrays indeed demonstrated the preference of *A. comosus* stem extract to cleave substrates after a pair of basic amino acids^29^. Orlandi-Mattos *et al*. highlighted the importance of performing further biochemical and pharmacological studies by using pure and well characterized components to clearly understand the biological effects of the commercially available *A. comosus* stem extract sold as stem bromelain^20^. These authors showed indeed that while low concentration of *A. comosus* stem extract mimics the prohormones convertases 1 and 2 cleavage pattern of proenkephalin, high bromelain concentration induces additional cleavage at non-specific sites. They attributed such difference in the cleavage pattern to the minor proteolytic constituent ananain, a cysteine protease with broad specificity. A dual action was also obtained with *A. comosus* stem extract on human plasma fibrin(ogen) and blood coagulation. While at low concentration a procoagulant effect was observed, an anticoagulant activity was shown at high concentration^30^. This paradoxical effect should be linked to the observation that while *A. comosus* stem extract administered orally showed effectiveness in reducing ecchymosis^31,32^, the assessment of the dose of this complex mixture to be used remains a critical factor.

Using purified and well biochemically and structurally characterized *A. comosus* stem extract proteases should thus provide the basis for a better understanding of their substrate specificity and inhibition profile as well as allow assessment of their in vitro cytotoxicity.

In the present work we determined the structures of bromelain (this term will refer to pure basic bromelain throughout the text) and ananain, both in their free forms and in complex with the inhibitors E64 and TLCK, and evaluated their cytotoxicity using adherent and non-adherent human cancer cell lines.

## Results

### Analysis of the purified ananain forms by mass spectrometry

Two forms of purified ananain were analyzed by ESI-Q-ToF MS: the oxidized and the *S*-thiomethylated forms (Supplementary Fig. 1). For the oxidized form three major peaks are detected: 23,455 Da, 23,473 Da and 23,616 Da (in decreasing order of intensity). The first one is best explained by an ananain that starts at Val1 like the available structure^33^ and finishes at Ser216, considering a catalytic Cys25 with a double oxidation (theoretical molecular weight (MW): 23,458.5 Da). The second peak would be identical to the first one but with a triple oxidation of Cys25 (theoretical MW: 23,474.5 Da). The third peak would correspond to an enzyme form with two additional amino acids (Gly217 and Pro218) with a double oxidation of Cys25 (theoretical MW: 23,612.7 Da). For the *S*-thiomethylated form the three major peaks are in decreasing order of intensity: 23,490.0 Da, 23,469.0 Da and 23,512.0 Da. The second peak best corresponds to a Val1-Ser216 form of ananain with a *S*-thiomethylated Cys25 (theoretical MW: 23,473.5 Da). The two other peaks would then be Na^+^ adducts (+ 23 Da and + 46 Da, respectively).

**Figure 1:**
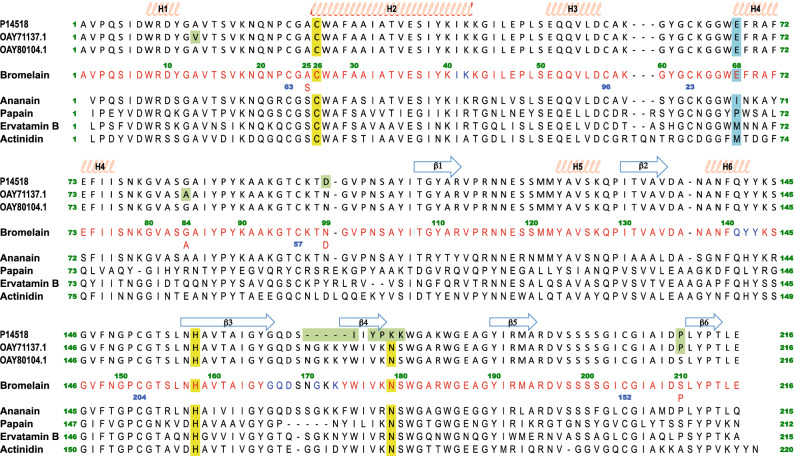
Structure based sequence alignment of bromelain with ananain (PDB: 6Y6L), papain (PDB: 1PPN), ervatamin B (PDB: 1IWD) and actinidin (PDB: 3P5U). Bromelain is also aligned with the three closest sequences identified in databases. The catalytic triad is highlighted in yellow, the Glu68 responsible for the bromelain specificity in light blue, amino acid difference between the database sequence and the structure in green. In the bromelain sequence, red amino acids are confirmed by MS and blue ones thanks to the electron density. Blue numbers below Cys residues indicate their disulfide bond partners.

### Analysis of the purified forms of bromelain by mass spectrometry

The ESI-Q-TOF MS analysis of the bromelain used for the crystallization reveals a significant heterogeneity of the sample, which could not be anticipated form SDS-PAGE pattern (Supplementary Figs. 2 and 3). The most intense peaks are gathered between 24,400.0 Da and 25,250.0 Da, with a maximum around 24,645.0 Da. This heterogeneity may have two origins; the heterogeneity of the amino acid sequence as previously reported^34^ as well as the heterogeneity resulting from the glycosylation pattern^1^. The sample was therefore further analyzed by ESI-Q-TOF MS/MS after digestion by trypsin. A sequence coverage of 94% (202 amino acids out of 215) was reached after several experiments (Fig. 1). Two different amino acids were identified at positions 25 (Ala or Ser), 84 (Gly or Ala), 99 (Asp or Asn) and 210 (Pro or Ser). The unidentified amino acids cluster in three regions: 41–42, 141–143 and 166–173. A NCBI blastp reveals a 100% sequence identity with OAY80104.1 sequence (Ser25, Gly84, Asn99 and Ser210). The second best hit, with 99.5% sequence identity, is OAY71137.1 (Ser25, Ala84, Asn99 and Pro210), but we do not have evidence of a valine at position 13 to fully match this sequence. Compared with the sequence P14518.1, which is annotated as stem bromelain, while the first two are referenced as ananain, we observe an insertion at position 169 and some substitutions in this loop as well as an Arg184Lys substitution not observed by MS.

**Figure 2:**
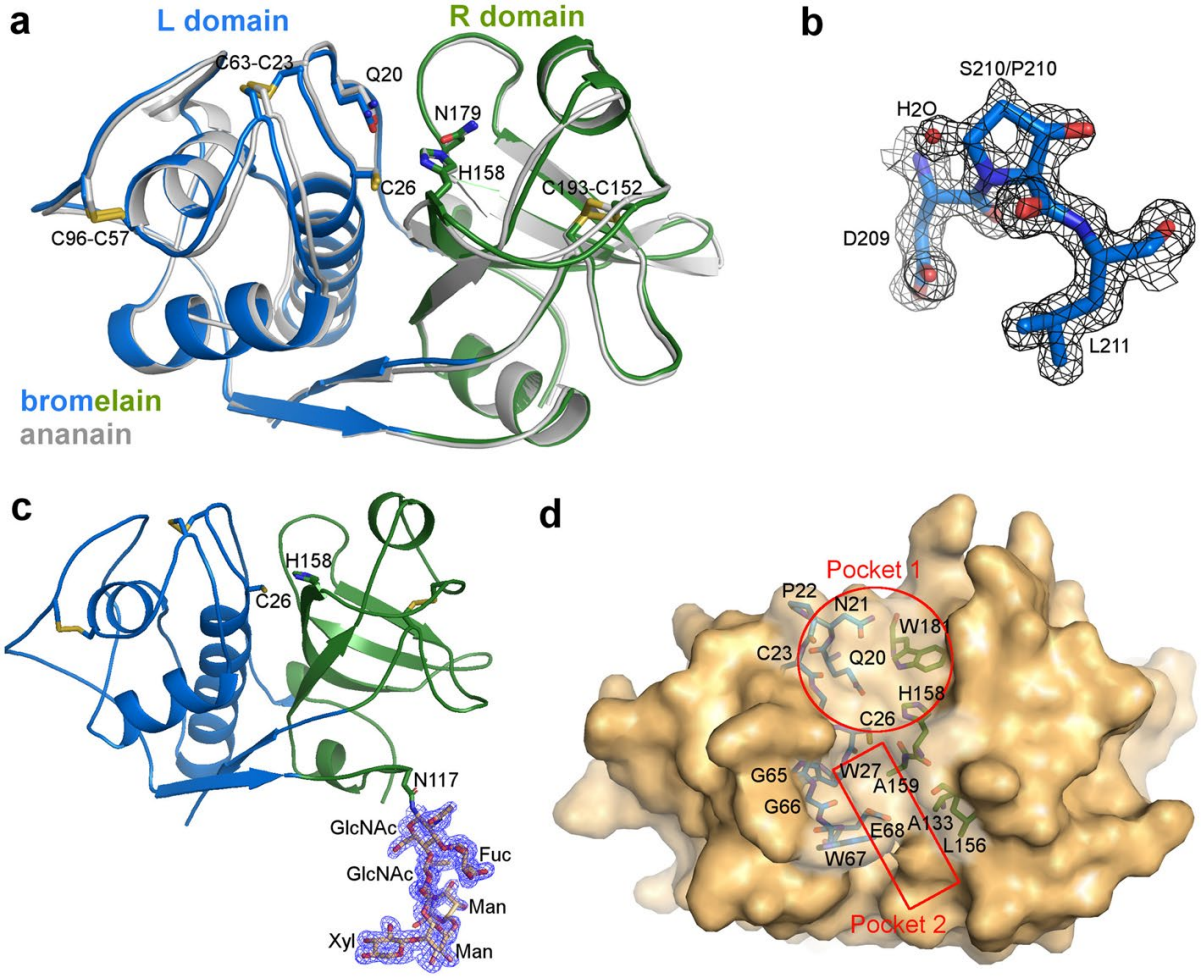
Overall structure of bromelain and ananain. (**a**) Cartoon representation of superimposed bromelain (L domain blue and R domain green) and ananain (gray) structures; important catalytic residues and disulfide bonds are represented as sticks. (**b**) Sticks representation of two different residues (serine and proline) in alternate conformations observed at position 210 of molecule B. 2Fo-Fc electron density map is displayed at a 1σ level. The water molecule shown is only concomitant with the S210 alternate conformation. (**c**) Sticks representation of the glycosylation of Asn117, 2Fo-Fc electron density map is also displayed at a 1σ level. (**d**) Surface representation of bromelain, residues forming pocket 1 and pocket 2 are shown as sticks in transparency.

**Figure 3:**
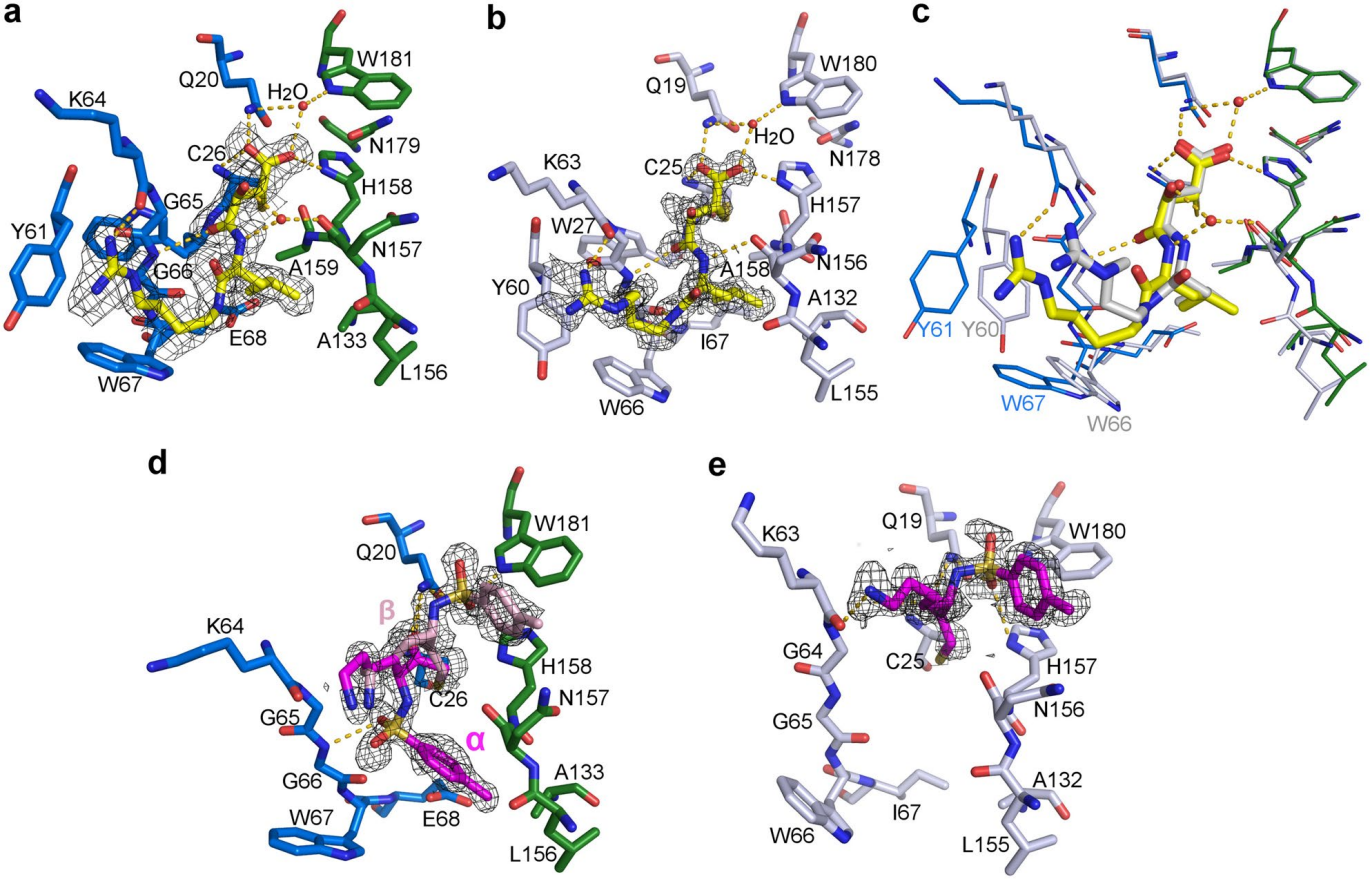
Structures of bromelain and ananain in complex with E64 and TLCK. (**a**) Residues from the active site surrounding of the bromelain:E64 complex, bromelain L domain in blue and R domain in green, and E64 in yellow; H-bonds are shown as dashed lines. The feature enhanced electron density map is displayed at a 1σ level around E64. (**b**) Same as (a) for the ananain:E64 complex (ananain in gray sticks). (**c**) Superimposition of the bromelain:E64 (blue, green and yellow) and ananain:E64 (gray) complexes. (**d**) Same as (a) for the monomer B of the bromelain:TLCK complex. The α and β alternate conformations of TLCK are respectively represented as magenta and light pink sticks. (**e**) Same as (d) for the ananain:TLCK complex (ananain in gray sticks).

The ESI-Q-TOF MS/MS experiments also allowed the identification of two forms for the glycosylation. The first one has a MW of 2368.6 Da (m/z = 1184.8, charge 2 +) identical to that identified by Ishihara *et al*. ((GlcNac)_2_(Fuc)_1_(Man)_2_(Xyl)_1_))^1^. Compared to the later, the second one has a MW of 2222.9 Da (m/z = 741.6, charge 3 +) and does not contain the fucose residue.

### Overall structures of ananain and bromelain

We obtained the crystallographic structure of ananain in its catalytic cysteine oxidized (ananain-SO_2_H) and *S*-thiomethylated (ananain-SCH_3_) forms at 1.25 Å and 1.30 Å resolution respectively, and bromelain with its catalytic cysteine *S*-thiomethylated (bromelain-SCH_3_) at 1.80 Å resolution. These ananain structures represent a significant resolution improvement compared to the recently released structure (pdb code 6OKJ, 1.73 Å)^33^, they do however not reveal new significant structural features.

All ananain structures belong to the P2_1_ space group with similar unit cell parameters (Supplementary Table 1) and an asymmetric unit containing two molecules. Likewise, all three bromelain structures belong to the C222_1_ space group, with similar unit cell parameters (Supplementary Table 1) and an asymmetric unit also containing two molecules.

Bromelain and ananain structures showed the typical overall fold observed in the C1A subfamily of cysteine proteases. The structures fold into two domains, the L and R domains. The active site is located in a cleft at the interface between the two domains. In bromelain, the L-domain, residues 11–112 and 209–216 is mainly formed by α-helices while the R-domain, residues 2–10 and 113–208 has predominantly an antiparallel β-sheet structure. The bromelain numbering will be used throughout the text, because of the extra N-terminal alanine in bromelain, the ananain numbering is always shifted by one unit toward lower value. The catalytic Cys26 and His158 are respectively located in the L and R domains (Fig. 2a). Gln20 and Asn179, which are thought to respectively contribute to the formation of the oxyanion hole and to form a hydrogen-bond with the protonated side chain of His158 for a favorable orientation of the imidazolium ring^35,36^, are also conserved in both proteases.

In each monomer of the ananain-SCH_3_ structure, the electron density of the Cys25 side chain extends to accommodate the thiomethyl moiety. This extension has been modeled with two alternative conformations and displays significant flexibility as indicated by the poor quality of the electron density (Supplementary Fig. 4c). In the bromelain-SCH_3_ structure, a single conformation is observed (Supplementary Fig. 4a) but the density indicates an occupation lower than 100% (refined at 30% in each monomer).

**Figure 4:**
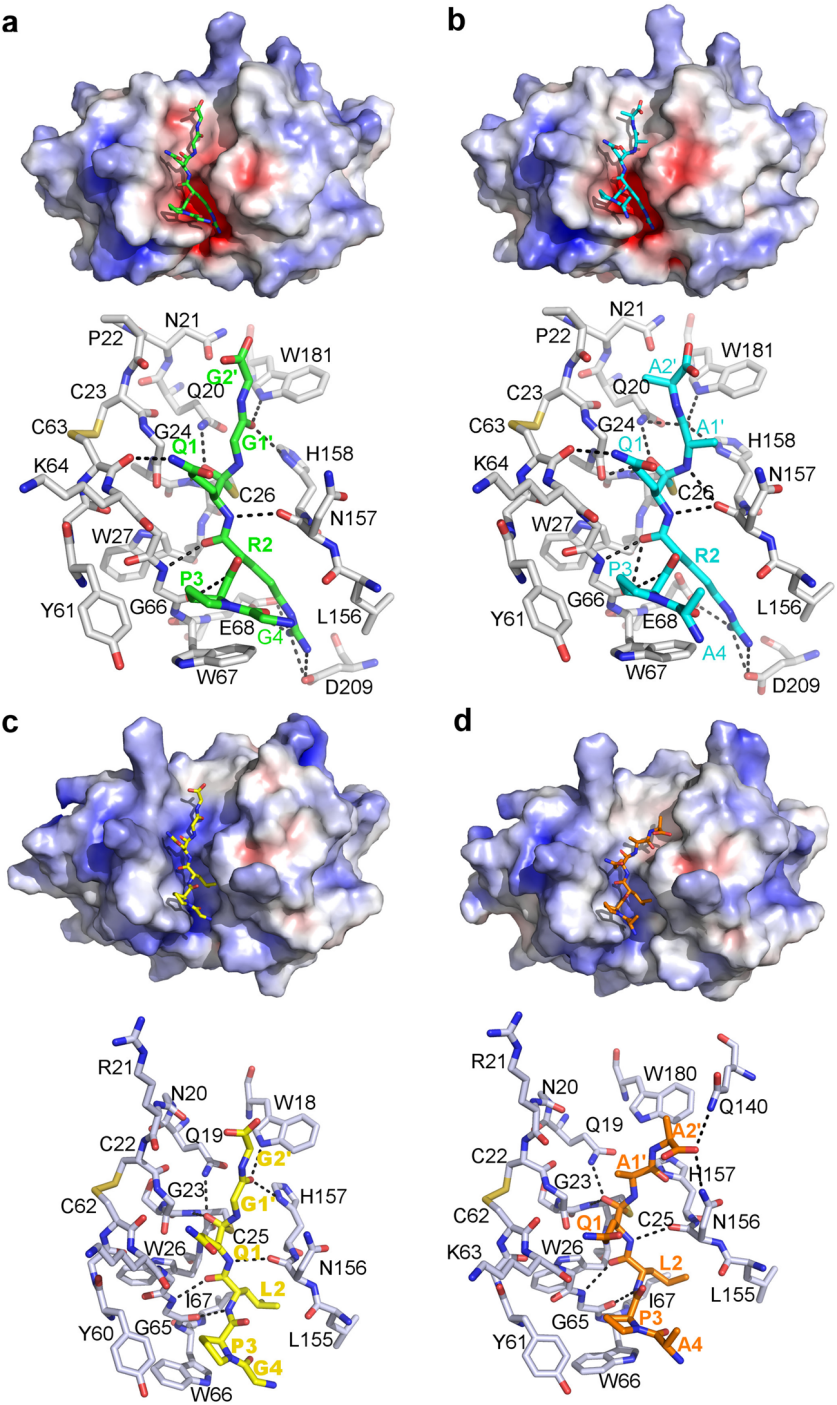
Peptide modeling in the active site of bromelain and ananain. For each model an electrostatic surface calculated with APBS with the peptide in sticks is shown, as well as a sticks representation of the active site surrounding with the H-bonds between the peptide and the protein (gray carbons) as black dashed lines. (**a**) Bromelain-GPRQGG model. (**b**) Bromelain-APRQAA model. (**c**) Ananain-GPLQGG model. (**d**) Ananain-APLQAA model.

In the ananain-SO_2_H structure, an electron density corresponding to catalytic cysteine with a double oxidation is well defined (Supplementary Fig. 4d) and confirms the results of Napper *et al*.^3^ OD1 interacts with the side chain of Gln19 and with the backbone N of Cys25, while OD2 interacts with the backbone N of Trp26. The third oxidation of Cys25 likely occurs much slowly due to a steric hindrance with the His157 side chain.

Three disulfide bridges, between Cys23 and Cys63, Cys57 and Cys96, and Cys152 and Cys204, were identified in the bromelain and ananain electron density maps (Fig. 2a). These residues are well conserved within the C1A subfamily of cysteine proteases^37^.

The MS analysis of bromelain identified four positions with two possible amino acids. At position 25 we could only identify the presence of the alanine in the electron density but not the serine. At position 84 an alanine is observed, but with a weaker density for its Cβ. Because the MS results confirmed that a glycine is also possible, we fixed the occupancy of both residues at 50% for refinement. At position 99, MS analysis indicates either an aspartate or an asparagine. The side chain of the amino acid at this position points toward the solvent and we cannot distinguish between the two possibilities. The structure therefore contains both residues with a 50% occupancy. At position 210, the structure clearly validates the two possible side chains (serine or proline), both residues were therefore included with a 50% occupancy in the structure (Fig. 2b). During the refinement procedure, the occupancy of these three residues was fixed while individual B factors were refined.

Bromelain is known to be glycosylated on Asn117. In monomer A, we observed almost the entire polysaccharide identified by MS: a GlcNAc linked to a second GlcNAc (β1–4) and a fucose (β1–3), a mannose linked to the second GlcNAc (β1–4) and to a xylose (β1–2). The second mannose molecule bound to the first one (β1–6) is not clearly identified in the electron density of the *S*-thiomethylated bromelain structure, but could be refined in the bromelain:E64 and bromelain:TLCK complexes (Fig. 2c). In monomer B, a single GlcNAc is observed in the electron density because the glycosylation is not stabilized in the crystal by a neighboring bromelain molecule, like in monomer A.

### Comparison of the active site cleft of ananain and bromelain with other cysteine proteases of the C1A subfamily

Yongqing *et al*.^33^ described two pocket-like structures (named pocket 1 and pocket 2) between the two domains of ananain. In bromelain, pocket 1 is formed by amino acids Gln20, Asn21, Gly24, Trp27, Gly65, Trp181 as well as the catalytic residues Cys26 and His158, and is strictly conserved in bromelain and other papain-like proteases, except for Asn21 that substitutes the usual glycine and points towards the center of the pocket (Fig. 2d). This substitution is unique among the C1A proteases structures analyzed (ervatamin B has a lysine at this position but pointing away from the catalytic residues). An effect on substrate specificity at positions P1′ and/or P2′ cannot be excluded. Pocket 2 is deep and narrow, and is made of the side chains of several hydrophobic residues, comprising Trp27, Trp67, Ala133, Leu156 and Ala159, as well as the negatively charged Glu68, which defines the bottom of the cavity and substitutes Ile67 in ananain (Fig. 2d). This pocket, known to correspond to subsite S2 in C1A cysteine proteases, is critical for the various substrate specificities observed for the members belonging to this subfamily of proteases^38^ and therefore named “substrate specificity pocket”^39,40^.

The S1 subsite lies in between these two pockets and is considered to be formed by Gly24, Cys26 and Gly65 that are highly conserved among C1A proteases. It is commonly believed that this subsite exerts relatively slight influence on substrate specificity but position 64, which is adjacent to this area, is more variable and could slightly tune the specificity at the P1 position. It is indeed a lysine in ananain and bromelain, an asparagine in papain and ervatamin B, and an aspartic acid in actinidin.

The S3 subsite, composed of residues Tyr60, Gly65 and Trp66 in ananain, is hydrophobic in nature and strictly conserved in bromelain, but the orientation of the tryptophan in bromelain (two conformations observed) is slightly affected by the different nature of the surrounding amino acids. An aromatic residue is also found at this position in papain (Tyr), ervatamin B (Trp) and actinidin (Phe) with slightly different orientations. On the other hand, while Gly65 is conserved in other C1A proteases, the position occupied by Tyr60 is more variable (Arg in actinidin and His in ervatamin B).

### Mode of binding of E64 to bromelain and ananain

E64 [L-trans-epoxysuccinyl-l-leucylamido-(4-guanidino) butane] is a naturally occurring cysteine proteases inhibitor isolated from cultures of *Aspergilus japonicus*^41^. It is well known to be a tight-binding inhibitor of the C1A proteases^42^. The structures of the bromelain:E64 and ananain:E64 complexes were obtained by soaking crystals of the *S*-thiomethylated form of the enzymes in a solution containing DTT and E64. The resolutions of these structures are respectively 1.85 Å and 1.30 Å (Supplementary Table 1). In the bromelain:E64 complex, a clear density corresponding to E64 bound to Cys26 is observed in each monomer (Fig. 3a). Within pocket 1, the two oxygen atoms of the carboxylic function of E64 make hydrogen bonds with the backbone nitrogen of Cys26, and the side chains of Gln20 and His158. The O4 carbonyl of the trans-epoxysuccinic acid moiety forms a hydrogen bond with Gly66 between pockets 1 and 2. The leucyl moiety is inserted into pocket 2, which is characterized by the presence of the Glu68 responsible for the unusual bromelain specificity at position P2. The subsequent repulsion induces a retreat of about 0.8 Å of Glu68 and the adjacent Trp67 compared to their positions in the free enzyme. Trp67 is part of the S3 subsite (along with Tyr61) that accommodates the guanidinobutane group and is not as well defined as in the free enzyme.

The resolution of the ananain:E64 structure is significantly higher than that of Yongqing *et al*. (1.3 Å vs 1.98 Å)^33^. The overall fold of the two structures is very similar, as well as the binding mode of E64, for which a clear electron density is observed in both monomers (Fig. 3b). A significant modification is however observed in subsite S3 of the 6MIS structure, where the side chains of Trp66 and Tyr60 are slightly pushed away from the catalytic center compared to their position in our ananain:E64 complex as well as compared to our free enzyme structure.

Interestingly, superposition of the ananain:E64 and bromelain:E64 complexes, also highlights differences regarding the S2 and S3 subsites. As expected, the E64 leucyl moiety is not as deeply inserted into the bromelain S2 subsite because of the presence of Glu68. In subsite S3, the shift of the Trp67 and Tyr61 in the bromelain:E64 complex compared to the ananain (free enzyme and in complex with E64) structures (Fig. 3c) as well as the free bromelain structure emphasizes the constrains of the accommodation of this inhibitor in bromelain and the plasticity of the S3 subsite.

### Mode of binding of TLCK to bromelain and ananain

TLCK (N^α^-p-tosyl-lysyl chloromethylketone) is known to inhibit both serine and cysteine proteases^14,43^ by forming covalent bonds with the catalytic serine and histidine residues in the serine proteases^44^, while a single covalent bond is observed with the catalytic cysteine in cysteine proteases^45,46^. We obtained the structures of ananain and bromelain in complex with TLCK at 1.34 Å and 1.45 Å resolution respectively (Supplementary Table 1), by soaking crystals of the *S*-thiomethylated forms in a high concentration of TLCK in the presence of DTT.

The overall structure of the ananain:TLCK complex doesn’t present significant modification compared to the free enzyme (root mean square deviation—rmsd —of 0.14 Å calculated for all Cα). In the active site of both monomers in the asymmetric unit, a density associated to the catalytic Cys25 clearly indicates a covalent bond with the chloromethyl carbon of TLCK (Fig. 3e) as observed in the papain^45^ and the lysine-specific gingipain complexes^46^, confirming thus the formation of only one covalent bond upon cysteine protease:TLCK complexes formation. The carbonyl oxygen O is H-bounded to the backbone nitrogen of Cys25 and NE2 of Gln19. The tosyl group of the inhibitor is well defined in the density, with O2S forming hydrogen bonds with His157 (ND1), Trp180 (NE1) and Gln19 (NE2), and the phenyl methane docked in the pocket formed by the side chain of residues Trp180, Gln140, Ala135, Ser136 and Asn156. The lysyl group is not as well defined in a position close to Gly23, Gly64 and Lys63. The general area of interaction of TLCK approximately corresponds to the S1′ subsite of the active site and is approximately similar to the orientation observed in the papain-TLCK complex^45^ except for the rotation of the tosyl group. This difference could be due to the position of Ala137 in the papain structure (Ser136 in ananain), which slightly reduces the pocket accommodating the phenyl methane moiety in ananain.

In the bromelain:TLCK complex, the two monomers present in the asymmetric unit must be distinguished. In monomer B, densities corresponding to two conformations of the inhibitor were identified. In conformation α (magenta sticks in Fig. 3d), the carbonyl oxygen O is H-bounded to the backbone nitrogen of Cys26 and NE2 of Gln20. The tosyl group of the inhibitor is well defined in the density, with O2S forming a hydrogen bond with the fucose of an adjacent molecule in the crystal, and the phenyl methane inserted in the S2 pocket. The lysyl group is not as well defined and is in a position close to Gly24, Gly65 and Lys64 like in the ananain:TLCK complex. In the conformation β (light pink sticks in Fig. 3d), which is equivalent to the one observed in the ananain:TLCK complex (Fig. 3e), the carbonyl oxygen as well as the lysyl group lie approximately at the same positions as in conformation α. The tosyl group lies however in a different region of the active site, with two hydrogen bonds involving O2S of TLCK (to NE1 of Trp181 and NE2 of Gln20) and the phenyl methane docked in the pocket formed by the side chain of residues Trp181, Gln141, Ala136 and Asn157. In this monomer we noticed a significant widening of the active site due to the displacement of the Tyr61-Trp67 loop of the L-domain (average displacement of about 1 Å for the Cα).

In monomer A, an electron density indicating a moiety covalently bond to Cys26 is also observed (Supplementary Fig. 4b). However, it could not be adequately interpreted neither by a TLCK molecule, which leads to several bad contacts with protein atoms, nor by S-thiomethylation, which only explains a portion of this density. In the absence of a proper identification of this moiety, we left this density unmodeled.

### Modeling of the proteases-peptide complexes

Because the unrestrained docking of peptides in the active site of proteases generates mostly irrelevant poses of the peptide, we chose to model the peptide in the tetrahedral transient form covalently bound to the catalytic cysteine that leads to the scissile bond cleavage. In this state, we forced a single bond between the carbon carbonyl of the scissile bond and the catalytic cysteine in its thiolate form, the carbonyl oxygen therefore becomes negatively charged, and we forced a protonated state for the catalytic histidine. The peptide is built in an extended conformation and the most common rotamers are selected. The peptide is then manually positioned without modifying any torsion angle to optimize the distance with the catalytic cysteine and minimize the clashes with the protein. The molecular dynamic refinement procedure implemented in YASARA is then applied in default setup (see “Materials and methods” for details).

For ananain, we started with the ananain-SCH_3_ structure and the PLQ tripeptide identified by Yongqing *et al*.^33^, extended by one and two glycines at the N- and C-terminal ends. A second modeling was also performed with the glycines substituted by alanines to probe the potential effect of side chains at these positions. The same procedure was used for bromelain. The bromelain:TLCK structure was chosen as starting model because of its higher resolution, and the GPRQGG and APRQAA peptides were used because of the similarity with ananain and the known preference of bromelain for a positive amino acid at P2. In each case, the orientation of the peptide in the 20 solutions generated was very similar. The solution with the best compromise between the highest structure quality Z-score, which includes dihedral angles, distance-dependent packing interactions and direction-dependent packing interactions, and the lowest energy was selected for analysis. The rmsd calculated for all the Cα between the refined protease-peptide complexes and the starting structures were 0.48 Å, 0.53 Å, 0.46 Å and 0.50 Å for ananain-GPLQGG, ananain-APLQAA, bromelain-GPRQGG and bromelain-APRQAA respectively. The peptides display a good shape and charge complementarity with the active sites (Fig. 4a–d).

In all models, the backbone of the P2 and P1 positions adopts a similar conformation with the same network of H-bonds involving the four polar atoms: P2-N is bond to the carbonyl oxygen of Gly66 (throughout the paragraph, remove one to obtain ananain numbering), P2-O to the nitrogen of the same residue, P1-N to the carbonyl oxygen of Asn158 and P1-O in the oxyanion hole formed by the backbone nitrogen of the catalytic cysteine and NE2 of Gln20 (Fig. 4a–d). Interestingly, this orientation of the peptide is very similar to the conformation of the leupeptin in complex with papain and falcipain-3^47,48^. The NE2 atom of the P1 Gln lies in the vicinity of the carbonyl oxygens of Cys63 and Lys64 only forming a clear H-bond with cysteine in the bromelain models. The P2 arginine side chain is bound to the side chains of Glu68 and Asp209 in bromelain. In ananain, the P2 leucine side chain lies in the hydrophobic S2 subsite. The P3 proline also adopts a conserved positioning in a shallow cavity formed by the Trp67 and Tyr61 side chains. The P4 Gly and Ala do not form significant interactions with the protease. The position of the P′ side of the peptide is not as well conserved (Fig. 4a–d). The carbonyl oxygen of P1′ forms H-bonds with the side chain nitrogens of Trp181 and His158 except for the ananain:APLQAA model were the interaction with the tryptophane is absent. The position of the P2′ residue varies in the different models. The absence of significant interactions of the first and last residues of the peptides with the proteins is clearly apparent in the graph displaying the root mean square fluctuation of the peptide residues calculated for 20 models generated by the molecular dynamic simulations (Supplementary Fig. 5d).

**Figure 5:**
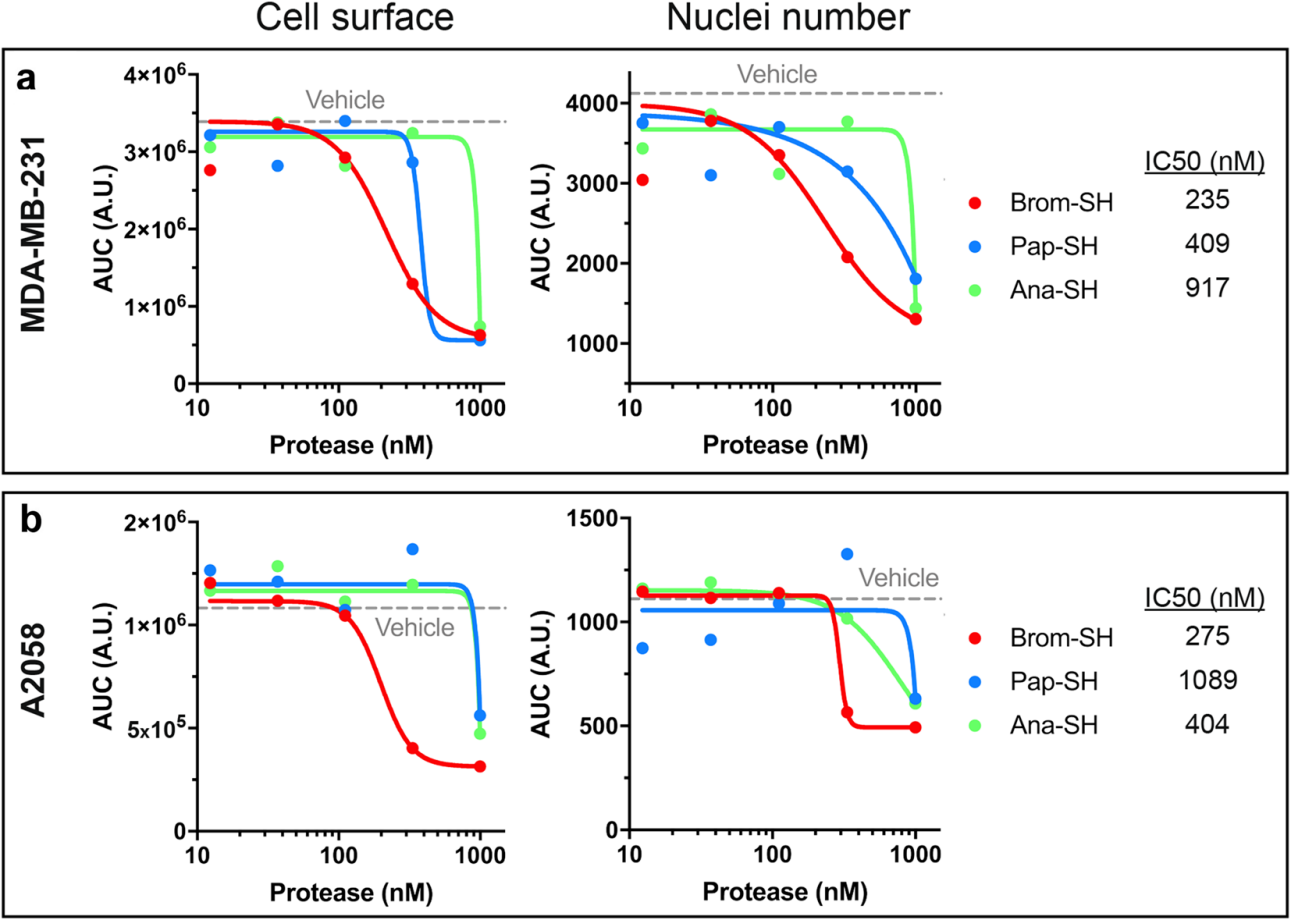
MDA-MB-231 (**a**) and A2058 (**b**) cells were treated during 72 h with increasing concentrations (12.33–1000 nM) of the active (-SH) form of bromelain, papain and ananain or vehicle only. Live cell imaging was used to measure the cell surface and the number of mKate2+ nuclei. The areas under the cell surface/nuclei number versus time curves (AUC) are plotted for each concentration tested. IC50 values are calculated based on the number of mKate2+ nuclei counted after 72 h of treatment.

### In vitro cytotoxicity test

The potential influence of bromelain, ananain and papain on the behavior of different human cancer cell lines was investigated by using live cell imaging. Two adherent cell lines (MDA-MB-231 cells: a triple-negative breast adenocarcinoma and A2058 cells: a metastatic melanoma) were used. The adherent cell lines were transduced in order to stably express a nuclear-targeted fluorescent protein (mKate2). The fluorescent nuclear label allows both a direct quantification of cell proliferation and a read-out for cell viability since the mKate2 label is lost during cell death.

As high cell density within an individual population can alter sensitivity to death inducing stimuli^49,50^, cells are seeded such that they are less than 50% confluent when first exposed to treatments. Following the addition of the proteases, live (mKate2 +) adherent MDA-MB-231 and A2058 cells within each population are counted using an automated high-throughput microscope housed within a tissue culture incubator. Phase contrast images are acquired in parallel to live cell counts to assess population confluence and morphology.

Adherent cancer cell lines were first treated with culture medium alone (vehicle control) or increasing concentrations of the active forms (where the catalytic cysteine is in its reduced form Cys-SH: protease-SH) of the three proteases. Cells were imaged every hour for 72 h starting 30 min after compound addition. Treatment with vehicle was not lethal to any cell line and the number of live mKate2+ cells and the surface area covered by cells increased exponentially during the 3 days of treatment (Supplementary Fig. 6a,b). When used at low concentrations (12.33–111 nM) bromelain-SH, papain-SH and ananain-SH had a minimal influence on the proliferation of the MDA-MB-231 and A2058 adherent cell lines (measured either by counting the number of mKate2+ cells or measuring the surface area covered by cells). A strong reduction of cell proliferation was observed with the three proteases used at 1 µM. In contrast with the two other proteases, bromelain-SH also inhibited cell proliferation when used at 333 nM (Fig. 5 and Supplementary Fig. 6). IC50 values for the active forms of the three proteases (Fig. 5) were calculated based on the number of mKate2+ nuclei counted at the end of the experiment (after 72 h of treatment).

**Figure 6:**
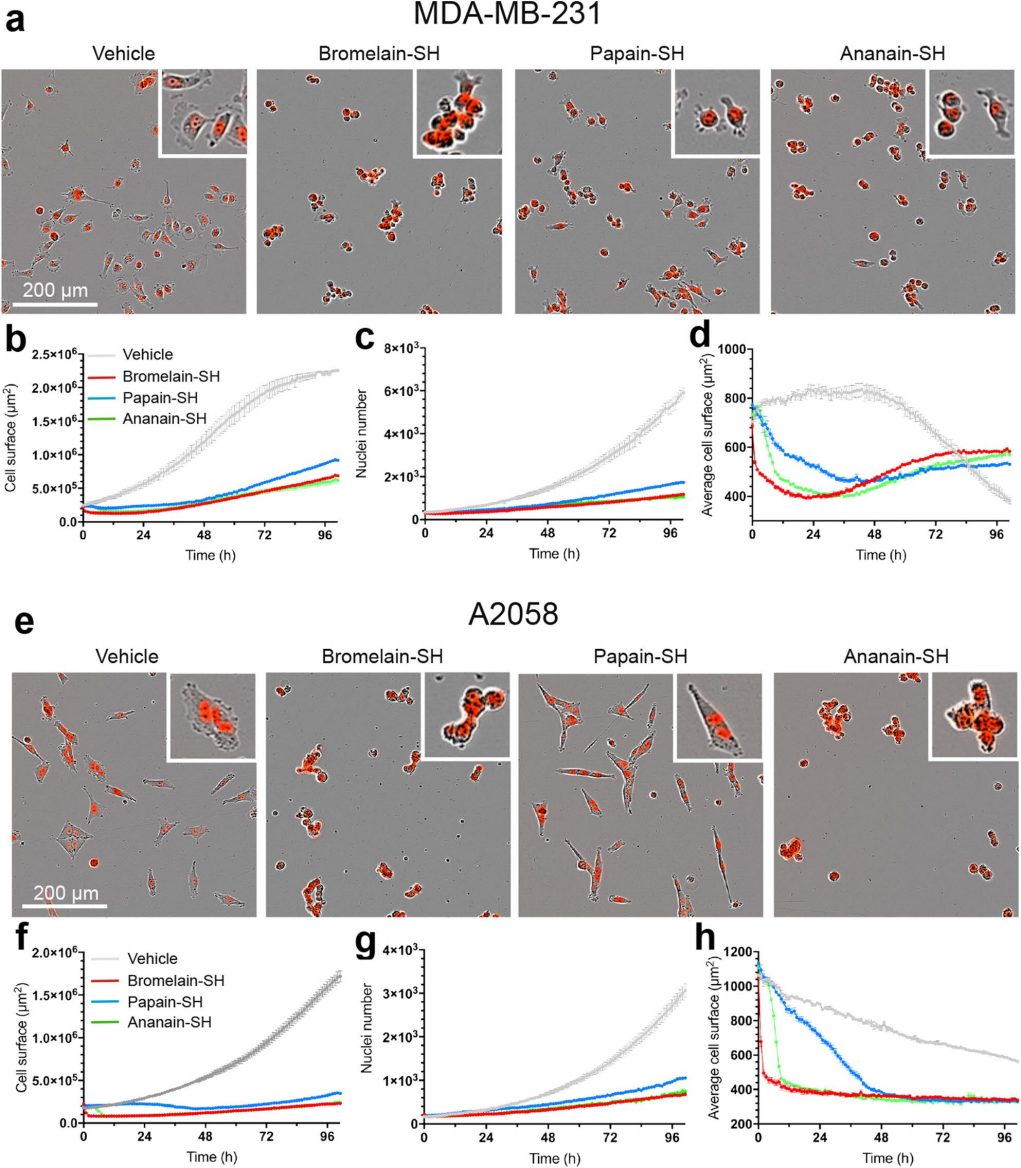
MDA-MB-231 (**a–d**) and A2058 (**e–h**) cells were treated during 100 h with the active (–SH) form of bromelain, papain and ananain (1 µM) or vehicle only. Live cell imaging was used to obtain pictures of the different culture conditions (**a** and **e**) and calculate the cell surface (**b** and **f**), the number of mKate2+ nuclei (**c** and **g**) and the average cell surface (**d** and **h**). Pictures were taken after 24 h of treatment. Phase contrast and mKate2 fluorescence images are merged. Scale bar = 200 µm. Data are presented as mean ± SEM (n = 3–4). Kolmogorov–Smirnov test: see Supplementary Table 2.

We next compared the behavior of the two cell lines exposed to either the active (Cys-SH) or inactive (Cys-E64) forms of the proteases. In that purpose, the cells were exposed to the proteases used at the highest concentration (1 µM). Live cell imaging revealed clear morphological alterations in response to these treatments. While vehicle-treated cells displayed a normal morphology characterized by a flat cytoplasm with active lamellipodial and pseudopodial extensions, the active proteases induced a rapid retraction of the cytoplasmic extensions, leading to the formation of loosely attached cell clusters in both cell lines (Fig. 6a, e and Supplementary Fig. 7). In agreement with the dose–response experiments (Fig. 5), the three proteases rapidly decreased the cell-covered area (Fig. 6b and f) and strongly reduced cell proliferation, as reflected by the very small increase in the number of mKate2+ nuclei throughout the treatment period (Figs. 6c and g). In order to better quantify the morphological alterations induced by these treatments, the average cell surface was calculated by dividing the cell-covered area by the number of nuclei (Figs. 6d and h). This analysis revealed clear differences in the kinetic of the cytoplasmic retraction. Bromelain-SH and ananain-SH both induced a very fast decrease of the average cell surface (even though the effect of ananain-SH was delayed by about 6 h). In contrast, papain-SH required a much longer exposure time (about 45 h) to induce a similar level of cytoplasmic retraction. A detailed microscopic observation of these cultures revealed the presence of a few dead cells in the A2058 but not in the MDA-MB-231 cells. The non-viable cells portrayed features of oncotic cell death with swollen cell bodies^51^. In contrast with the active forms, the inactive proteases (Cys-E64) did induce neither cytoplasmic retraction, nor decreased cell proliferation (Supplementary Figs. 8 and 9).

**Figure 7:**
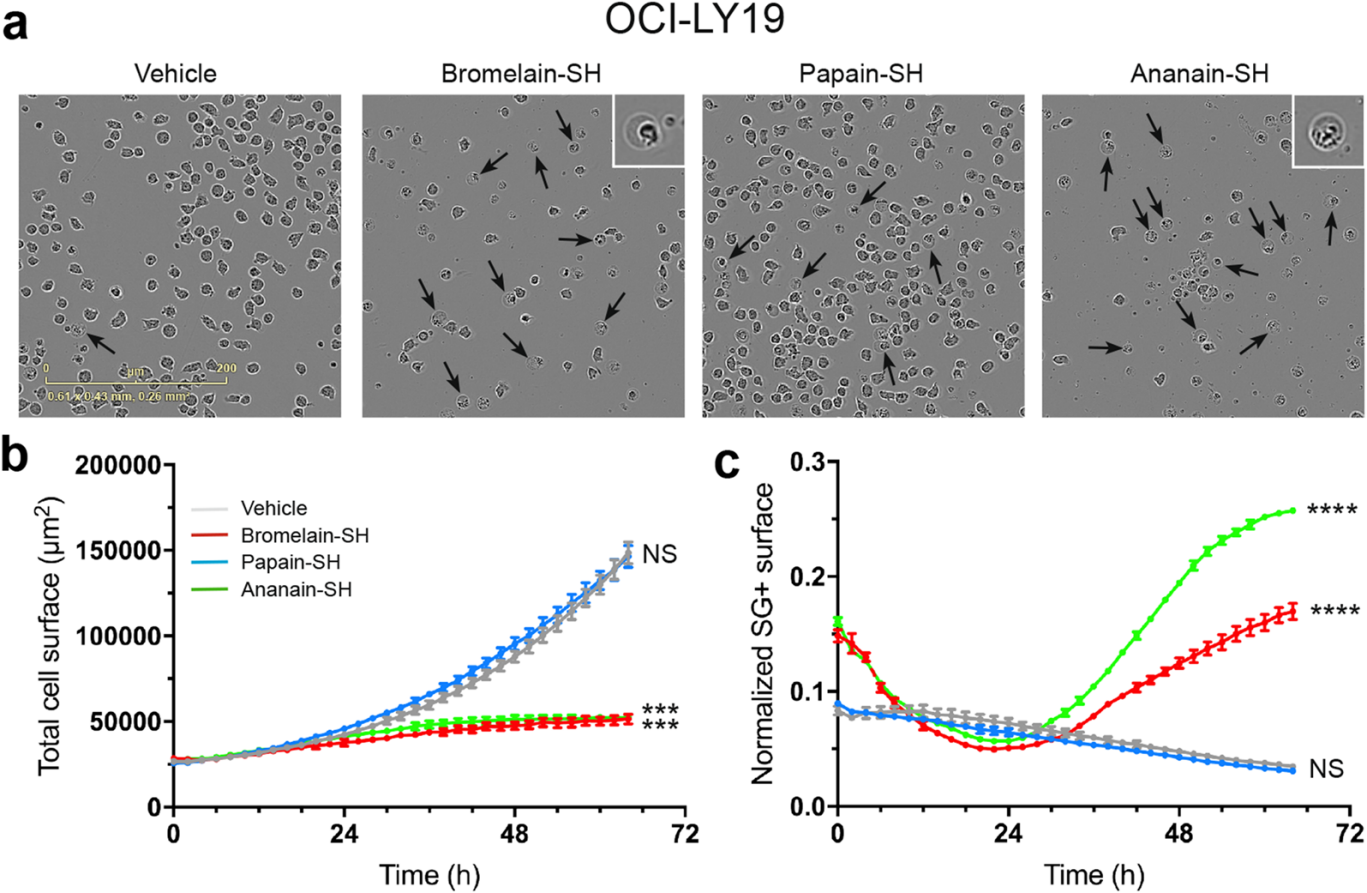
OCI-LY19 cells were treated during 64 h with the active (–SH) form of bromelain, papain and ananain (1 µM) or vehicle only. Live cell imaging was used to obtain pictures of the different culture conditions (**a**) and calculate the total cell surface (**b**) and the normalized SYTOX Green (SG) positive cell surface (**c**). Pictures were taken after 64 h of treatment. Arrows indicate dead cells. Scale bar = 200 µm. Data are presented as mean ± SEM (n = 3–4). Kolmogorov–Smirnov test: NS, not significant; ***p < 0.001; ****p < 0.0001.

**Figure 8:**
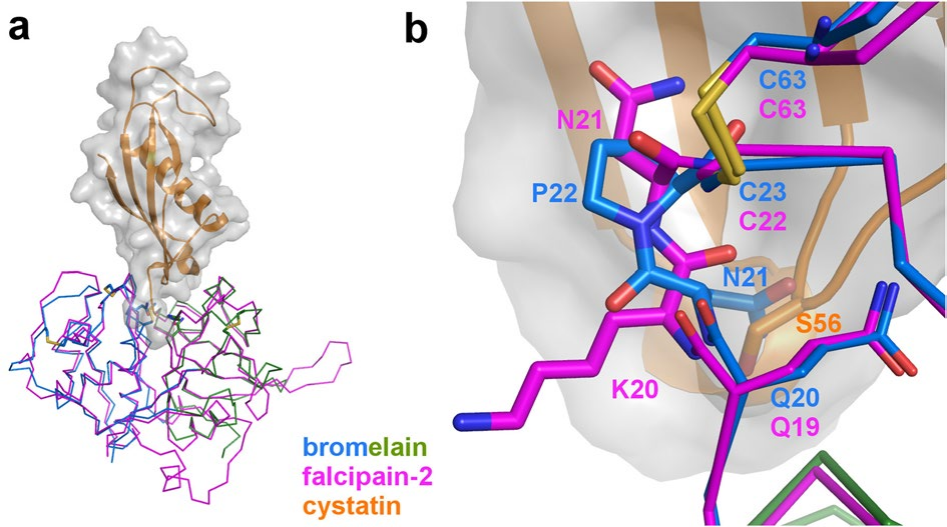
Bromelain-cystatin interaction. (**a**) Superimposition of the falcipain-2(magenta):cystatin(orange and gray transparent surface) complex on the bromelain (L domain blue and R domain green) structure. (**b**) Close up view of the N21-P22 *cis* peptide likely interfering with cystatin binding while adjacent residues are well superimposed with falcipain-2.

Collectively, these observations suggest that the active forms of the three proteases prevent an increase in cell population through an anti-proliferative effect rather than by inducing cell death.

In order to evaluate whether the anti-proliferative effect of the active proteases was related with their capacity to induce cell retraction from the substrate and cell rounding, non-adherent OCI-LY19 and HL-60 cells were used. Due to the absence of mKate2 nuclear tag in these cells, cell death was evaluated by measuring the SYTOX Green (SG) fluorescence (which only labels cells with compromised plasma membrane integrity) following exposition to either the active or inactive forms of the proteases. The OCI-LY19 cells were exposed to the active and inactive forms of the three proteases (1 µM) and both total cell surface and normalized SG+ cell surface were recorded as a function of time by using live cell imaging. While the surface occupied by cells increased exponentially in a similar way in vehicle- and papain-SH-treated cells, bromelain-SH and ananain-SH both prevented this increase (Fig. 7a,b). A similar inhibitory profile was also observed with HL-60 cells (Supplementary Fig. 9). This inhibition of cell growth was associated with a striking increase of SG + dead cells (Fig. 7a and c). Most dying cells were characterized by the formation of single blister (Fig. 7a, insets) characteristic of oncotic cell death. In contrast with the active forms, the inactive proteases (Cys-E64) did affect neither the total cell surface nor the normalized SG+ cell surface (Supplementary Fig. 11).

## Discussion

### Overall structures of ananain and bromelain

Thanks to the purification protocol previously described to separate the cysteine proteases present in the *A. comosus* stem^14^, we were able to obtain three structures of bromelain and four ananain structures at a resolution higher than 2 Å. The overall fold is well conserved, with rmsd calculated for all Cα pairs between 0.07 and 0.62 Å. This finding also shows that the glycosylation identified by MS as well as observed in some monomers of the bromelain structures does not impact the protein structure. Because it is located away from the active site, this glycan moiety is not expected to have any effect on the enzyme activity and specificity (Fig. 2c). The possible effect of glycosylation on the stability of this enzyme was however not assessed.

The bromelain preparation used presents the unusual characteristic of having amino acid heterogeneities at least at the positions 25, 84, 99 and 210, as identified by MS and confirmed by the structures for residues 84 and 210. For residue 99 it is indeed not possible to distinguish between the electron density of an aspartic acid or an asparagine at the resolution obtained for our structures. For position 25, if present in the structure the serine (instead of an alanine) identified by MS would have been observed in the electron density. This indicates that at least three (iso)forms of bromelain are present in the sample submitted to crystallization: the one corresponding to the OAY80104.1 GenBank reference (Ala25, Gly84, Asn99 and Ser210), a second one not identified in the databases with at least Ala84 and Pro210 (+potentially Asp99), and one not identified in the structures and containing a serine at position 25 (+potentially other variations). The Protein Data Bank recently released a structure of the recombinant bromelain precursor (PDB code 6U7D). This structure contains a 100 amino acid N-terminal extension responsible for the inhibition of the proteolytic activity. The overall fold of the part common with the active protease presented in this report is very similar, with a rmsd of 0.67 Å calculated for all common Cα.

### Comparison of the ananain and bromelain complexes with E64

The *k*_2_ values for inactivation of papain, ananain and bromelain by the specific and irreversible cysteine proteases inhibitor E64 are 6.3 10^5^, 2.5 10^5^ and 5.1 10^2^ M^−1^ s^−1^, respectively^9,52^. Despite this 1000 fold difference, the orientation of the E64 molecule in the bromelain:E64 complex is very similar to the one observed in C1A proteases such as ananain and papain, and most of the hydrogen bonds stabilizing these complexes are conserved. The main factor responsible for the lower affinity of E64 for bromelain is the repulsion between the leucyl moiety of E64 and the Glu68 side chain present at the bottom of the S2 subsite instead of a isoleucine in ananain (Fig. 3a,b) or another hydrophobic residue in most C1A proteases, preventing the deep insertion of the inhibitor. The guanidinobutane group of E64 is located in the S3 subsite formed by Tyr61 and Trp67 (bromelain numbering). The significant displacement of these residues compared to the free protease (also observed in the bromelaine:TLCK complex) as well as the two conformations observed for Trp67 side chain in the bromelain-SCH_3_ structure are indications of a significant flexibility of the S3 subsite. This flexibility is further confirmed by the modeling of the protease-peptide complexes mimicking the tetrahedral transition state for which this region displays the largest displacement in the active site vicinity compared to the starting structure.

### Comparison of the S1-S3 subsites of ananain and bromelain with other representative members of the C1A subfamily

Several studies explored the specificity of ananain and bromelain compared to other C1A proteases over the years. Rowan *et al*.^13^ used a series of peptidyl-NH-methyl coumarin substrates, Gosalia *et al*.^29^ used solution-phase substrate microarrays representing a 722-member library of fluorogenic protease substrates having the general format Ac-Ala-X-X-(Arg/Lys)-coumarin (where X corresponds to all natural amino acids except cysteine) and Choe *et al*.^28^ used a combinatorial peptide library composed of a total of 160,000 tetrapeptide substrate sequences completely randomizing each of the P1, P2, P3, and P4 subsites with 20 amino acids. Napper *et al*.^3^ used a library of 400 dipeptides. Except for Rowan *et al*.^13^ and Napper *et al*.^3^, which purified the ananain and bromelain, all other above-cited studies used a commercially available stem bromelain mixture that mostly represents the activity of purified bromelain. The latter indeed accounts for about 90% of the total proteolytic fraction while ananain only represents about 9%^3,9^. In addition, Yongqing *et al*.^33^ studied the specificity of purified ananain with a tripeptidyl substrate library (REPLi) containing a total of 512 tripeptide pools covering 3375 individual peptides.

As described in the results section, the S1 subsite is well conserved in C1A proteases family. The residues usually identified as preferred at this position are Arg, Lys, Gln and to a lesser extent Thr. This could appear as in disagreement with the REPLi results of Yongqing *et al*.^33^ because Ile/Leu were identified at this position for several of the 20 best peptide pools, but the identification of the cleaved bond was only performed by MS on the best peptide. Indeed, the presence of three glycines before the variable tripeptide and two glycines after may lead to multiple positioning of the peptide in the active site and thus multiple cleavage possibilities. When the Ile/Leu is identified at the P1 position of the peptide pool, these amino acids likely occupies the S2 site and the cleavage occurs between the last two glycines of the peptide substrate. The presence of Arg/Lys at the P2 position in the REPLi results is a clear indication of this phenomenon as it is concomitant with Ile/Leu at the P1 position and ananain has been shown to have a very weak activity against substrate having a basic amino acid at the P2 position^2,9,13,14^. According to our modeling results, the preference of bromelain and ananain for a residue with a long H-bond donor side chain is likely due to the presence of two main chain carbonyl in the P1 vicinity (Cys23 and Lys64 in bromelain, Fig. 4). Because it involves main chain functions (one from a conserved disulfide bond), this feature is also present in most C1A proteases.

The S2 subsite is clearly known as the main determinant of the substrate specificity and most of the C1A proteases favor the cleavage of peptides characterized by a hydrophobic residue at this position^13,28,29,33,37,38,53–55^. The deep pocket forming S2 is defined by the side chain of residues Trp26, Trp66, Ile67, Ala132, Leu155 and Ala158 in ananain. The specific discrimination for hydrophobic residues at the P2 position (Leu/Ile for ananain vs Phe for papain) is induced by substitutions maintaining the hydrophobic nature of the pocket (e.g. in papain Trp26, Tyr67, Pro68, Val133, Val157 and Ala160). With respect to this feature, bromelain clearly stands out. It is indeed characterized by the sole Ile67/Glu68 substitution in this pocket compared with ananain, but the specificity is dramatically modified toward an arginine at this position^3,28,29^. More specifically, a 250 fold increase of the purified bromelain specific activity for a Arg-Arg containing substrate and a 90 fold decrease for Phe-Arg containing substrate were observed compared to the purified ananain^13^. The presence of Asp209 in bromelain (Asp208 in ananain and Ser205 in papain), which was identified as a ligand of the P2 arginine by modeling, also influences the specificity of the S2 subsite. This position is equivalent to Glu245 in cathepsin B, which has been described as responsible for an improved hydrolysis of peptide with an arginine at the P2 position and a concomitant decrease of affinity for peptide with a hydrophobic side chain^56^. However, for cathepsin B, the level of discrimination between positive and hydrophobic residues at position P2 remains significantly lower than that of bromelain.

Unlike for the P1 and P2 subsites, the P3 subsite has formerly not won much interest, essentially due to the lack of a method to systematically compare all amino acids combinations. Furthermore, the exhaustive analysis conducted by Choe *et al*.^28^ revealed that there is subtle yet interesting diversity among P3 specificities among the proteases of C1A subfamily, such as preference for basic amino acids, aliphatic amino acids, or proline, even if it is not as apparent as P2 specificity. Interestingly, these authors noticed a preference for substrates with proline at the P3 position for bromelain and the same was found for ananain^33^. According to modeling this specificity is driven by the conformations of Tyr61 and Trp67, which form an ideal shallow pocket to accommodate the kinked peptides containing a P3 proline (Fig. 4a–d). Analysis of the P4 subsite shows that there is not much specificity at this position as confirmed by our modeling, which didn’t reveal any specific interaction involving this position.

### Electrostatic effect of the bromelain S2 subsite specificity on small inhibitors

Ritonja *et al*.^16^ determined the second-order rate constants for inactivation of papain by iodoacetate and iodoacetamide to be 1422 and 84 M^−1^ s^−1^, respectively. The corresponding values for bromelain were 17.7 and 5.5 M^−1^ s^−1^, demonstrating, in contrast to the archetypal member of the C1A subfamily, a much lower reactivity. These values likely reflect the repulsion of negatively charged inhibitors because of the apparent negative charge of the catalytic cavity due to the presence of Glu68 as emphasized by the difference in electrostatic surface calculated for bromelain and ananain (Fig. 4a–d).

### Weak inhibition of bromelain by cystatins compared to most members of C1A proteases subfamily

Cystatins are highly efficient proteinous inhibitors of the C1A cysteine proteases. For example, the *K*_i_ values for inhibition of ananain and papain by chicken cystatin were found to be 1.1 10^–9^ and < 5 10^–12^ M, respectively^9^.

The structures of the papain:stefin B (human) and of the falcipain:cystatin (chicken) complexes reveal three conserved structural elements in the cystatin family responsible for the tight binding with proteases^57,58^: (i) the N-terminal end of cystatin, which targets the active site with a conserved Gly9-Ala10 di-peptide preceded by a residue fitting into the S2 site (Leu8 in chicken cystatin), and (ii and iii) the 53QLVSG57 and 103PWL105 hairpins of cystatin, which interact with the shallow conserved pocket 1 of the protease active site.

The inhibition of bromelain by chicken cystatin is on the other hand much weaker, with a *K*_i_ value of 3.6 10^–5^ M^9^. The superimposition of bromelain to the falcipain-2:cystatin complex (40% amino acid sequence identity) reveals two elements explaining this *K*_i_ value. First, the Leu8 residue of cystatin does not display the highest affinity for the S2 subsite that is known to favor positively charged residues. Second, the interaction with the 53QLVSG57 hairpin is affected at the Ser56 level by the original positioning of Asn21 (Fig. 8a,b). Indeed, the side chain of Asn21 is oriented in the opposite direction compared to the equivalent residue in other C1A proteases because of the unique presence of a proline at position 22, which induces a *cis* conformation of the Asn21-Pro22 peptide bond. The conformation of Trp67 (in bromelain) potentially interfering with the binding of the N-terminal end of cystatin, was also pointed out. However, the fact that this residue seems flexible and is conserved in ananain, which is efficiently inhibited by cystatin, means that this residue is likely not a key determinant for this inhibitory process.

Because the interactions of the two hairpins are conserved features of the mode of action of cystatins, the poor inhibition of bromelain by chicken cystatin is very likely a general characteristic of this protease. Other proteases have also been described for being poorly inhibited by cystatins, but the structural reasons behind^59^ are different than the ones identified in bromelain.

### In vitro cytotoxicity of purified cysteine proteases is due to protease activity

The goal of our cytotoxicity assay was to study the effect of individual purified and fully active proteases on the survival and proliferation of adherent and non-adherent cell lines, instead of the *A. comosus* stem extract usually used. This extract is indeed a complex mixture containing essentially various cysteine proteases, for which the oxidation state and therefore the active proteolytic fraction is not controlled.

Our results obtained with purified and fully active bromelain, ananain and papain show a strong reduction of cell proliferation (with MDA-MB231 and A2058 cancer cell lines) at a concentration of 1 μM (≥ 0.33 μM for bromelain). Control experiments with irreversibly inhibited proteases had no cytotoxic effect, clearly emphasizing the importance of the catalytic activity. Proteases are thought to non-specifically cleave adherence proteins, leading to the release of the cells from the substratum and a consequential cytoskeleton perturbation responsible of the arrest of cell division. Similar results were observed with *A. comosus* stem extracts^60^ and the C1A cysteine protease fastuosain^61^.

### What makes bromelain and ananain different than other C1A cysteine proteases?

The anti-inflammatory action of *A. comosus* stem extract has been linked to the substrate specificity of the protease^20^. In contrast, both ananain and bromelain had a strong effect on the proliferation of the OCI-LY19 and HL-60 non-adherent cell lines while papain, the archetypal member of the C1A subfamily, had none. This result is particularly surprising because the substrate specificity of ananain, with a preference for a hydrophobic residue at P2 is more similar to that of papain, which also prefers a hydrophobic residue at this position, than to that of bromelain, which prefers a positively charged residue. This leads to the paradoxical conclusion that the effect of ananain and bromelain on the proliferation of the non-adherent cell lines tested is due to their proteolytic activity, but not linked to the substrate specificity. More intriguing, these two proteases are characterized by a feature yet unidentified not shared by the closely related papain. A better stability and activity of these proteases in the culture medium could explain this result, but this medium is similar to the one used for adherent cell lines (10% serum), in which papain is active. The inhibition profiles of ananain and bromelain (e.g. by cystatin and iodoacetate) are also clearly different, and only bromelain is glycosylated. The most common feature between ananain and bromelain remains their 77% sequence identity, which could lead to a yet unidentified targeting of the proteases to specific location on the cell surface.

At this stage, it remains impossible to understand at the molecular level the numerous effects of *A. comosus* stem proteases, highlighting the need for more exhaustive studies with other cell lines in combination with proteomics tools to identify the specific proteins targeted by these proteases.

## Materials and methods

### Purification of *Ananas comosus* stem proteases

*Ananas* *comosus* stem proteases were purified as described previously^14^. Briefly, stem bromelain powder was suspended in acetate buffer 100 mM, pH 5.0 before being subjected to centrifugation. The supernatant constituting the total protein soluble fraction was applied onto an SP-Sepharose Fast Flow (13 × 2.5 cm internal diameter) home-made column pre-equilibrated with 100 mM sodium acetate buffer, pH 5.0. The unbound material was washed away with ten column volumes of the pre-equilibrating buffer and elution of the bound proteins was performed with a linear concentration gradient of sodium acetate buffer, pH 5.0. The chromatographic fractions were assayed for amidase activity using DL-BAPNA as a substrate as previously described^62^. The chromatographic fractions were pooled according to their amidase activity profile and separately subjected to chemical derivatization (*S*-thiopegylation) with a thiol-specific polyethylene glycol derivative, methoxy polyethylene glycol ortho-pyridine disulfide (mPEG-OPSS), to allowing their efficient chromatographic separation from the irreversibly oxidized protein material. Finally, the pegylated proteases (enzymatically active species) were converted into their *S*-methylthio-derivates by reduction with DTT and then reaction with an excess of *S*-methyl methanethiosulfonate (MMTS) to selectively and reversibly block the cysteine proteases, preventing their autolysis and/or irreversible oxidation of their catalytic cysteine residues. The resulting reaction mixture was applied to a SP-Sepharose Fast Flow column. After a washing step, the bounded proteins were eluted with a linear gradient of sodium acetate buffer^14^. The final chromatographic step of the *S*-thiomethylated bromelain and ananain forms, as well as their purity, assessed by SDS-PAGE, are presented in Supplementary Fig. 3. To carry out the crystallization screening, the fractions corresponding to bromelain and ananain were pooled and concentrated to the desired volume by ultrafiltration (AMICON system; membrane cutoff 3.0 kDa) and on a 5000 MWCO VIVASPIN 15R concentrator. For all samples, the buffer was exchanged with water after exhaustive washing on the VIVASPIN device. The protein samples were kept at − 20 °C until use.

To perform the cytotoxicity tests, *S*-thiomethylated forms of papain (prepared according to Azarkan *et al*.^62^) bromelain and ananain were activated in the presence of DTT. The samples were then separated into two aliquots. The aliquot corresponding to the active protease was exhaustively dialyzed against water to eliminate the excess of DTT and immediately stored at − 20 °C until use. To obtain the irreversibly inactivated proteases, the specific inhibitor E64 was added to the second aliquot and the reaction was conducted under moderate stirring until the residual proteolytic activity (against Z-Arg-Arg-AMC for bromelain and Z-Phe-Arg-AMC for ananain and papain) was undetectable. The sample was exhaustively dialyzed against water to eliminate the excess of E64 and DTT and immediately stored at − 20 °C until use.

### Mass spectrometry analysis

The whole protein analysis was performed by ESI-Q-TOF MS (electrospray-ionization quadrupole time-of-flight mass spectrometry) in positive ion mode (WATERS, MICROMASS). The protein samples were at a protein concentration of 10 µM, 30% ACN, 0.5% formic acid (final) in ammonium acetate 25 mM. Spectra are displayed as the mass to charge ratio (*m/z*). Calibration was performed using clusters of phosphoric acid in *m/z* range 90–3000, corresponding to raw spectra acquisition range. Resolution obtained was 8600 and mass accuracy is 100 ppm. The *max ent1* method was used for deconvolution of the spectra.

For the sequence analysis, the samples were digested with a cocktail of proteases before being reduced and alkylated. The digested protein samples were analyzed by LC–ESI–MS/MS. Spectra were analyzed with Data analysis 4.0 (BRUKER). MASCOT SERVER 2.2.04 and PROTEIN SCAPE 3.0 (BRUKER) were used for database searches. Carbamidomethyl of cysteines and oxidations of methionine were set as variable modifications. A quality test was run in parallel using a BSA sample to monitor the entire process.

### In vitro cytotoxicity test

#### Cell lines

Human MDA-MB-231 and A2058 cell lines were maintained in high glucose Dulbecco's modified Eagle's medium (DMEM) supplemented with 10% (v/v) fetal bovine serum (FBS), 100 IU/mL penicillin, 100 μg/mL streptomycin, 1 mM sodium pyruvate and 2 mM glutamine. Non-adherent OCI-LY19 cells were cultured in RPMI 1640 medium supplemented with 10% (v/v) FBS, 100 IU/mL penicillin, 100 μg/mL streptomycin and 1 mM sodium pyruvate. All culture reagents were purchased from Invitrogen (Merelbeke, Belgium). Cultures were maintained in humidified tissue culture incubators (HERACELL, THERMO SCIENTIFIC) at 37 °C with 5% CO_2_ and 95% air. All cultures were mycoplasma free as confirmed by the MYCOALERT detection kit (LONZA).

#### mKate2 expressing cell lines

Gene transfer lentiviral plasmid pLV SV40 NLS mKate2 (NeoR) was purchased to E-ZYVEC (Villeneuve d’Ascq, France). This plasmid allows mKate2 fluorescent protein with a Nuclear Localization Signal and selection marker expression driven by SV40 or PGK promoter respectively.

Lentiviral vectors were generated by the GIGA Viral Vectors platform (University of Liège). Briefly Lenti-X 293 T cells (CLONTECH, 632180) were co-transfected with a pSPAX2 (ADDGENE, Cambridge, MA, USA) and a VSV-G encoding vector^63^. Viral supernatants were collected 48 h, 72 h and 96 h post transfection, filtrated (0.2 µM) and concentrated 100× by ultracentrifugation. The lentiviral vectors were then titrated with qPCR lentivirus titration (TITER) kit (ABM, LV900, Richmond, BC, Canada)^64^.

MDA-MB-231 and A2058 cells were transduced with the lentiviral vector (70 TU/cell). Transduced cells were selected with neomycin/G418/geneticin (CAYLA/INVIVOGEN, ant-gn-1) (3 mg/mL for MDA-MB-231, 1 mg/mL for A2058) and fluorescence activated cell sorting (FACSAria III, GIGA-Flow Cytometry platform, University of Liège). The absence of RCL and mycoplasma in cell supernatant was confirmed with qPCR lentivirus titration kit and MYCOALERT PLUS mycoplasma detection kit (LONZA, LT07-710) respectively.

#### Cell seeding and compound addition

Experiments were performed in Falcon 96-well plates (Corning). Twenty-four hours before the start of the treatments, adherent cell lines (MDA-MB-231 and A2058) grown in 10 cm standard tissue culture dishes (FALCON, BECTON-DICKINSON) were trypsinized and counted. 100 µL of cell suspension containing 2000 MDA-MB-231, 1500 A2058 and 6000 OCI-LY19 viable cells was added manually to 96-well tissue culture plates. OCI-LY19 cells were seeded in poly-l-ornithine (SIGMA) coated 96-well plates. Test compounds were prepared in growth medium as threefold concentrated solutions, filter sterilized on 0.22 µm MILLEX-GV membranes (MERCK MILLIPORE) and 50 µL of these solutions were added to the cell cultures. In some experiments, the culture medium was supplemented with 20 nM SYTOX Green (SG) nucleic acid stain (THERMOFISHER).

#### Acquisition of population images

Cell population images were obtained over time using an INCUCYTE S3 dual color live content imaging system (ESSEN BIOSCIENCES, Welwyn Garden City, UK) residing within an IN-VITROCELL ES NU-5831 (NUAIR) tissue culture incubator maintained at 37 °C with 5% CO_2_. Images were acquired using a 10 × objective lens in phase contrast and red fluorescence (Ex: 565–605 nm, Em: 625–705 nm) channels for the adherent cell lines and in phase contrast and green fluorescence (Ex: 440–480 nm, Em: 504–544 nm) channels for the non-adherent cell line. At least four images were acquired from each well every 60–120 min for a maximum of 100 h. All INCUCYTE experiments were performed at least in triplicate.

#### Live cell counting and cell surface measurements

Automated image analysis routines were optimized for each cell line using the IncuCyte S3 software package and training data from vehicle and compound-treated samples. Images were analyzed using a routine with the following settings to count mKate2+ objects (Segmentation: Top Hat; Parameter adaption, radius: 100 µm; threshold adjustment: 0.5; Edge split on; Edge sensitivity − 5) and SG + objects (Segmentation: Top Hat; Parameter adaption, radius: 100 µm; threshold adjustment: 2.0). Surface area covered by cells was measured using the INCUCYTE CONFLUENCE software (Segmentation adjustment, background: 0.4/0.1; Cleanup, hole-fill: 200/0 µm^2^, adjust size: 0/−1 pixels; Filter, minimum area: 250/90 µm^2^, maximum eccentricity: 0/0.9995 for adherent/non-adherent cell lines, respectively). mKate2+ were expressed as objects per image, cell surface and SG+ area measures, expressed as µm^2^ per image, were then analyzed further on EXCEL (MICROSOFT) and GRAPHPAD PRISM 7. The average cell surface area of adherent cell lines was calculated by dividing the cell surface area by the number of mKate2+ nuclei. Normalized SG+ surface was calculated by dividing the SG+ cell surface area by the total cell surface area. Counts more than tenfold different than the one immediately preceding in the time course were censored. The observed distribution of two groups was compared by using the Kolmogorov–Smirnov test in PRISM 7. *P*-values lower than 0.05 were considered as significant. IC50 values were calculated by using the IC50 calculator from AAT BIOQUEST (https://www.aatbio.com/tools/ic50-calculator/).

### Crystallization

All crystals used were prism-shaped and obtained within two weeks using the sitting drop vapor diffusion method at 293 K. The ananain with its *S*-thiomethylated catalytic cysteine was concentrated to 20 mg/mL in water and the precipitant solution contained sodium–potassium tartrate 1 M and MES buffer 0.1 M at pH 6.0. The crystals used for the ananain:E64 and ananain:TLCK complexes were grown in a drop containing the same protein solution and 0.5 M Li_2_SO_4_ in a 0.1 M citrate buffer at pH 5.6. They were then soaked for 20 min in a 6 μL drop of the precipitant solution complemented with 0.2 μL of 1 M DTT and 0.1 μL of 50 mM E64 in 50% ethanol or 0.2 μL of 1 M TLCK solubilized in 100% DMSO. The ananain-SO_2_H crystal was obtained using a 13 mg/mL protein solution and a precipitant solution containing 1 M Li_2_SO_4_ and 0.5 M (NH_4_)_2_SO_4_ in a 0.1 M citrate buffer at pH 5.6. All the ananain crystals used for the data collection were soaked in a cryoprotectant solution containing 1.8 M ammonium sulfate and 45% glycerol before freezing in liquid nitrogen.

Bromelain solution was concentrated to 17 mg/mL in water and the precipitant solution containing 20% PEG 4000 and 20% 2-propanol in a 0.1 M citrate buffer at pH 5.5 for bromelain-SCH_3_ and pH 6.0 for the bromelain:E64 and bromelain:TLCK. For the two complexes, crystals were soaked in a 4 μL drop of precipitant solution complemented with 0.2 μL of DTT and 0.2 μL of the same E64 and TLCK stock solutions as for ananain, but the soaking times were respectively 30 and 50 min. The cryoprotectant solution for the bromelain crystals was made of PEG 6000 20% and glycerol 40%.

### Data collection, structures solution and determination

The diffraction data were collected on the Proxima1 beamline of the Soleil synchrotron. The data were indexed, integrated and scaled using XDS^65^. The crystals used for the bromelain and ananain structures belong respectively to the orthorhombic C222_1_ and monoclinic P2_1_ space groups. All structures were solved by molecular replacement with Phaser^66^ using the structure of ervatamin B as search model (pdb code 1IWD)^67^ for the bromelain-SCH_3_ structure and the structure of bromelain-SCH_3_ for the ananain-SCH_3_ structure. The refinement and model building cycles were respectively performed with PHENIX.REFINE (ananain)^68^ or REFMAC5 (bromelain)^69^ and COOT^70^. A summary of the relevant statistics of the data collection and refinement is given in Supplementary Table 1. The figures were prepared using PYMOL (The PYMOL Molecular Graphics System, Version 2.3.2 Enhanced for Mac OS X, SCHRÖDINGER, LLC.).

The coordinates and structure factors of the structures have been deposited in the Protein Data Bank with the PDB ID codes 6YCE (bromelain-SCH_3_), 6YCF (bromelain:E64), 6YCG (bromelain:TLCK), 6Y6L (ananain-SCH_3_), 6YCB (ananain-SO_2_H), 6YCC (ananain:E64) and 6YCD (ananain-TLCK).

### Modeling of the peptide in the active site of the proteases

The modeling of the peptides in the active site of ananain and bromelain was performed with the molecular dynamic refinement macro of YASARA^71^. The structures were prepared by removing all solvent molecules as well as the alternate conformations. The highest resolution structures were chosen (bromelaine:TLCK and ananain-SCH_3_). The md_refine macro performs a steepest descent minimization, a simulated annealing of the solvent, a local steepest descent minimization without electrostatics to remove bumps, a second simulated annealing with all the atoms followed by 500 ps of molecular dynamic simulation. Twenty evenly spread snapshots are then further refined and the models evaluated. The Yasara2 forcefield was used. The goal of using molecular dynamics simulations to search conformational space is to try to avoid the minimization to get trapped in local minima, thereby raising the hopes for a significantly better result^71^. Supplementary Fig. 5 presents the energy and Z-score of each model as well as the root mean square fluctuation (RMSF) of the main chain atoms of all the residues present in the simulations. Supplementary Fig. 5c,d clearly highlight a similar RMSF profile for bromelain and ananain, as well as for the peptides.

## Supplementary information

Supplementary Information.
