# Supplementary material for: Structures of the free and inhibitors-bound forms of bromelain and ananain from Ananas comosus stem and in vitro study of their cytotoxicity
Source: Sci Rep. 2020 Nov 11;10:19570. doi: 10.1038/s41598-020-76172-5 (PMC7658999; doi:10.1038/s41598-020-76172-5)
Supplement: Supplementary file 1 — Supplementary Information. [file 41598_2020_76172_MOESM1_ESM.pdf]

Structures of the free and inhibitors-bound forms of bromelain and ananain from *Ananas comosus* stem and *in vitro* study of their cytotoxicity

Mohamed Azarkan*[1], Erik Maquoi[2], François Delbrassine[3], Raphael Herman[3], Nasiha M'Rabet[1], Rafaèle Calvo Esposito[1], Paulette Charlier[3], and Frédéric Kerff*[3].

[1] Laboratoire de Chimie Générale (Unité de Chimie des Protéines), Faculté de Médecine, Université Libre de Bruxelles, Campus Erasme (CP 609), B-1070 Bruxelles, Belgium

[2] Laboratoire de Biologie des Tumeurs et du Développement, GIGA-Cancer, Université de Liège, B-4000 Liège, Belgium

[3] UR InBioS, Centre d'Ingénierie des Protéines, Université de Liège, B-4000 Sart Tilman, Liège, Belgium

Running Title: Ananain and bromelain structures and cytotoxicity.

* Corresponding Authors:

Dr. Mohamed Azarkan, address above, telephone +322 555 67 84; e-mail: mazarkan@ulb.ac.be; Fax: +32 2 555 67 82

Dr. Frédéric Kerff, address above, telephone +3243663620; e-mail: fkerff@uliege.be; Fax: +3243664954

keywords: *Ananas comosus*, bromelain, ananain, cysteine protease, cytotoxicity

Supplementary Table 1. X-ray data collection and refinement statistics.

	**Bromelain-SCH_3**	**Bromelain:E64**	**Bromelain:TLCK**	**Ananain-SCH_3**	**Ananain-SO_2H**	**Ananain:E64**	**Ananain:TLCK**
PDB code	6YCE	6YCF	6YCG	6Y6L	6YCB	6YCC	6YCD
**Data Collection:**							
Wavelength	0.97911	0.97934	0.97934	0.98011	0.98011	0.98011	0.98011
Space group	C 2 2 21	C 2 2 21	C 2 2 21	P21	P21	P21	P21
a, b, c (Å)	91.3, 137.7, 82.0	90.3, 140.5, 84.2	91.21, 137.23, 82.60	34.5, 57.9, 119.9	34.4, 58.1, 119.8	34.3, 59.5, 119.1	34.4, 58.4, 119.2
α, β, γ (°)	90, 90, 90	90, 90, 90	90, 90, 90	90, 93.0, 90	90, 93.5, 90	90, 92.9, 90	90, 92.6, 90
Resolution range (Å)[a]	52.7 - 1.8 (1.84 - 1.8)	45.4 - 1.83 (1.88 - 1.83)	52.8 - 1.45 (1.53 - 1.45)	41.61 - 1.3 (1.37 - 1.3)	41.8 - 1.25 (1.28 - 1.25)	42.05 - 1.3 (1.37 - 1.3)	41.76 - 1.34 (1.42 - 1.34)
Rmerge (%)[a]	7.4 (105.8)	10 (187)	7.1 (49.3)	8.0 (65.8)	7.6 (217)	10.6 (58.3)	5.5 (59.9)
<I>/<σI> [a]	19.2 (1.8)	14.6 (0.9)	17.9 (4.3)	11.2 (1.2)	12.2 (0.8)	8.3 (1.3)	15.7 (2.6)
Completeness (%)[a]	99.4 (92)	98.2 (77.6)	100.0 (99.8)	97.1 (94.9)	96.7 (89.1)	97.7 (91.3)	99.2 (96.0)
Redundancy [a]	12.7 (10.9)	12.5 (9.4)	11.7 (10.4)	6.8 (6.7)	6.6 (5.5)	6.2 (5.1)	6.5 (5.4)
**Refinement:**							
Resolution range (Å)	52.7 – 1.8	41.6 – 1.85	45.62 – 1.45	39.9 - 1.3	39.9 - 1.26	34.3 - 1.3	39.7 - 1.35
No. of unique	47750	39374	87043	112107	123322	114950	103368
R work (%)	17.0	17.0	11.5	12.0	12.3	14.4	11.9
R free (%)	20.6	20.7	15.5	14.0	15.1	16.8	14.9
No. Atoms							
Protein	3387	3361	3402	3405	3378	3398	3356
Water	328	371	440	473	461	447	449
Other	76	150	168	66	63	88	61
RMS deviations from ideal stereochemistry							
Bond lengths (Å)	0.009	0.009	0.017	0.011	0.007	0.008	0.016
Bond angles (°)	1.50	1.66	1.93	1.21	0.96	1.11	1.41
Mean B factor (Å2)							
Protein	23.9	27.6	17.3	17.2	13.5	16.1	17.1
Water	35.0	37.8	33.4	31.7	26.9	29.5	32.2
Other	32.6	39.7	26.5	37.9	26.5	25.5	26.9
Ramachandran plot:							
Favoured region (%)	98.3	97.9	98.1	98.3	98.6	98.6	98.1
Allowed regions (%)	1.7	2.1	1.9	1.7	1.4	1.4	1.9

[a] Numbers in parenthesis refer to the highest resolution shell.

Supplementary Table 2. Statistical analysis of Figure 6**.**

		Vehicle	Bromelain-SH	Papain-SH	Ananain-SH
Cell surface	Vehicle		****	****	****
	Bromelain-SH	****		****	NS
	Papain-SH	****	****		****
	Ananain-SH	****	NS	***	
Nuclei number	Vehicle		****	****	****
	Bromelain-SH	****		***	NS
	Papain-SH	****	***		**
	Ananain-SH	****	NS	****	
Average cell surf.	Vehicle		****	****	****
	Bromelain-SH	****		****	****
	Papain-SH	****	****		***
	Ananain-SH	****	*	****	
		MDA-MB-231			A2058

The observed distribution of two groups was compared by using the Kolmogorov-Smirnov test. NS: not significant; * p<0.05; ** p<0.01; *** p<0.001; **** p<0.0001.
Blue and green shaded cells correspond to the p-values for MDA-MB-231 and A2058 cells, respectively.

Supplementary Figure 1.

a

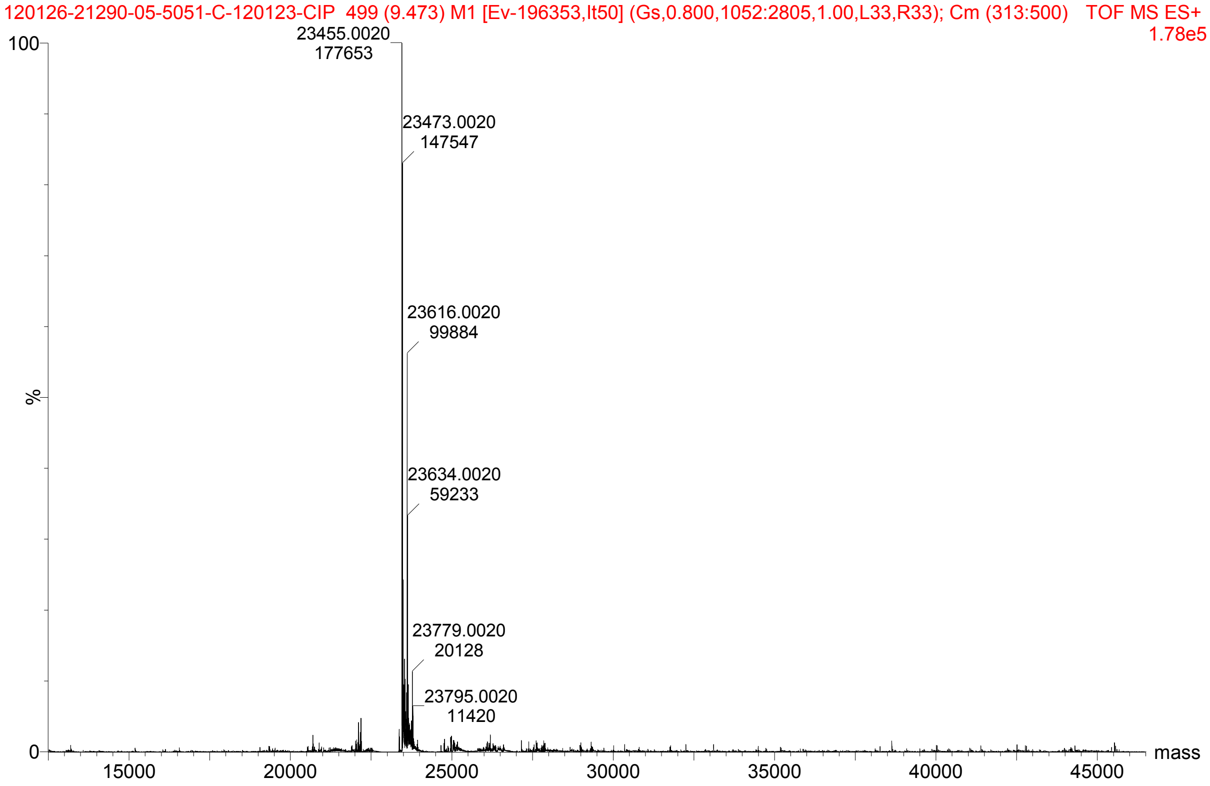

b

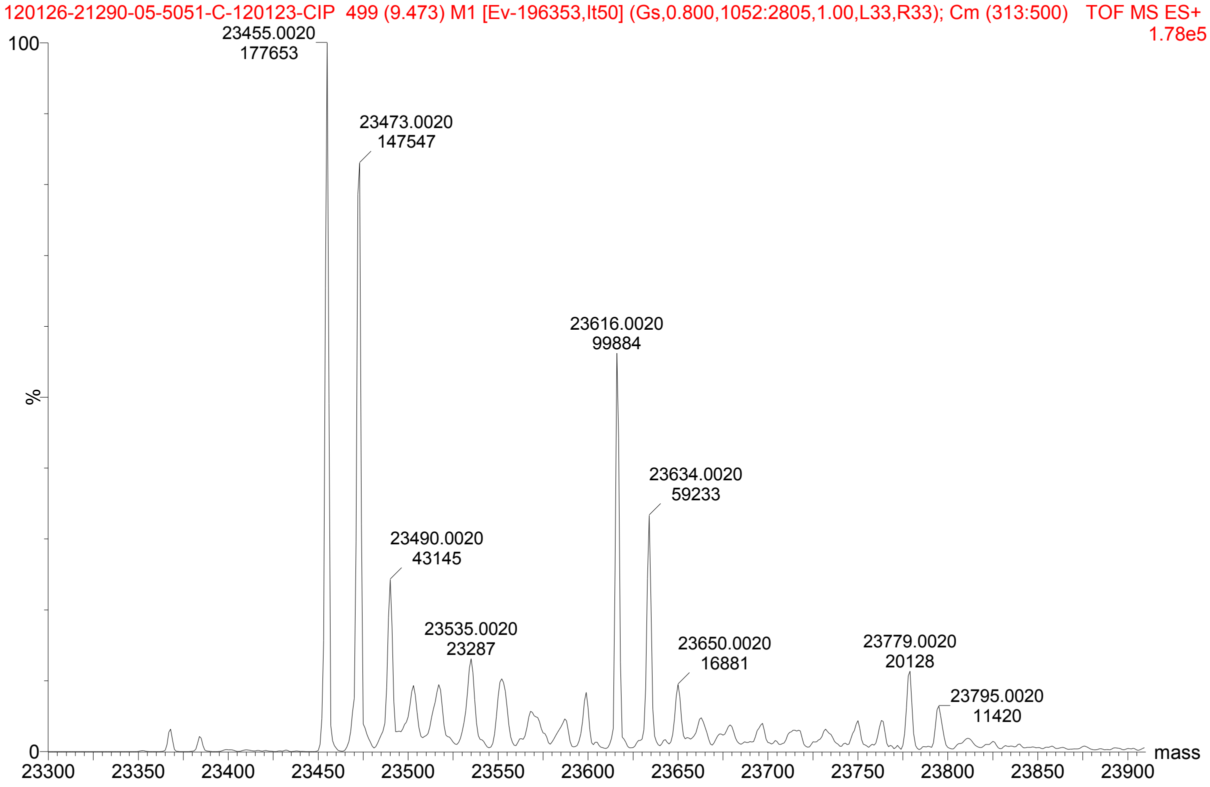

Mass spectrometry spectrum of the S-thiomethylated ananain sample, broad spectrum (**a**) and zoom on the major peaks (**b**).

Supplementary Figure 2.

a

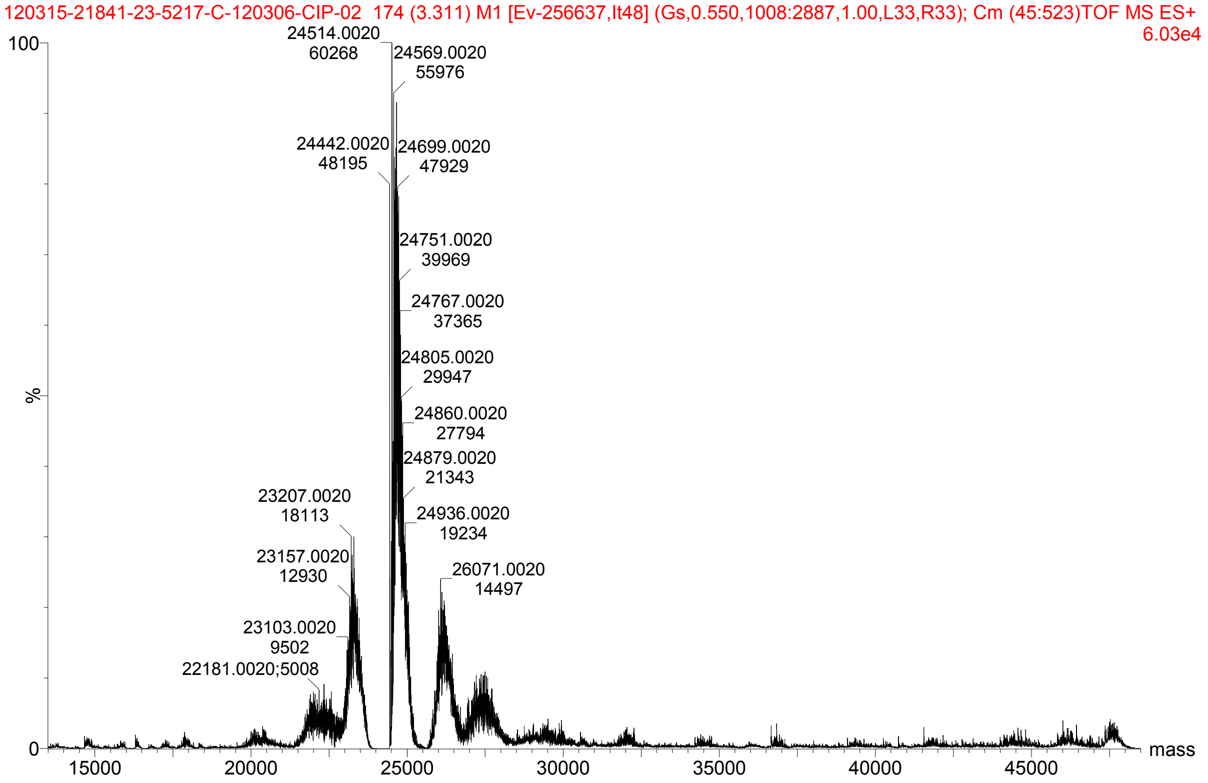

b

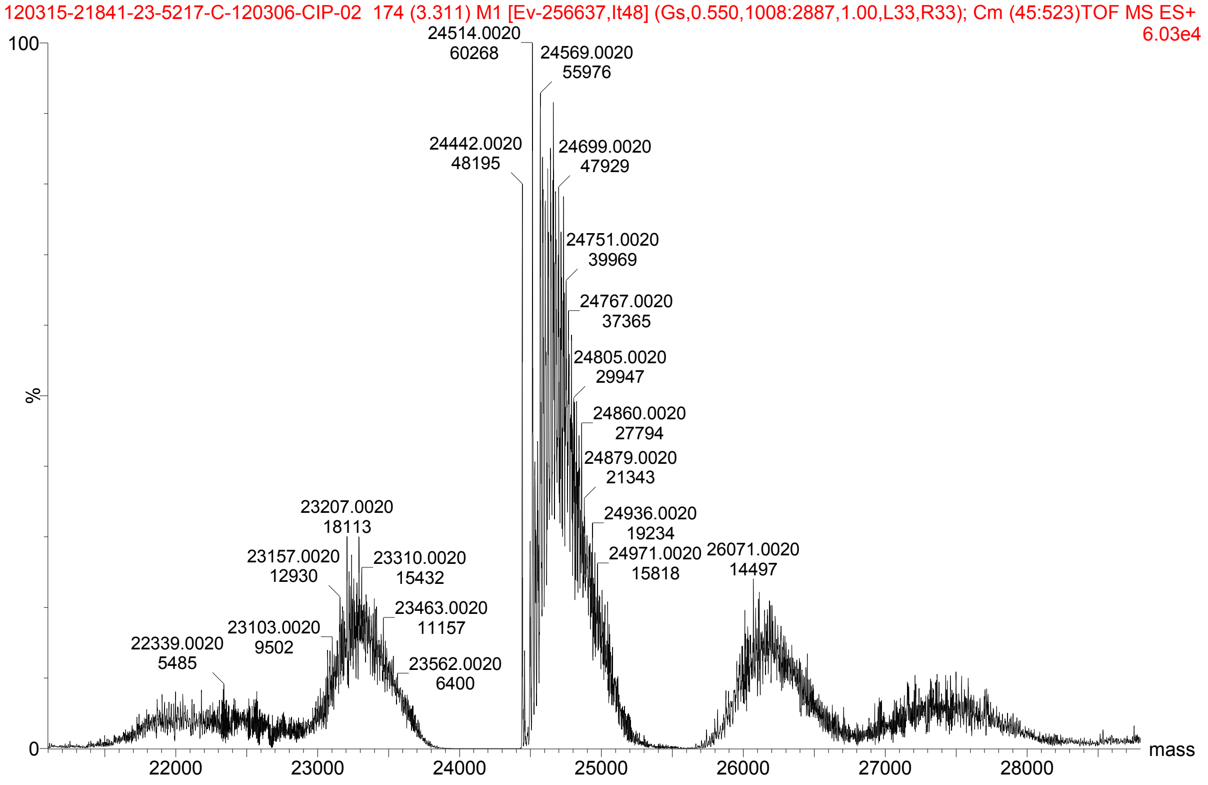

Mass spectrometry spectrum of the S-thiomethylated bromelain sample, broad spectrum (**a**) and zoom on the major peaks (**b**).

Supplementary Figure 3.

a

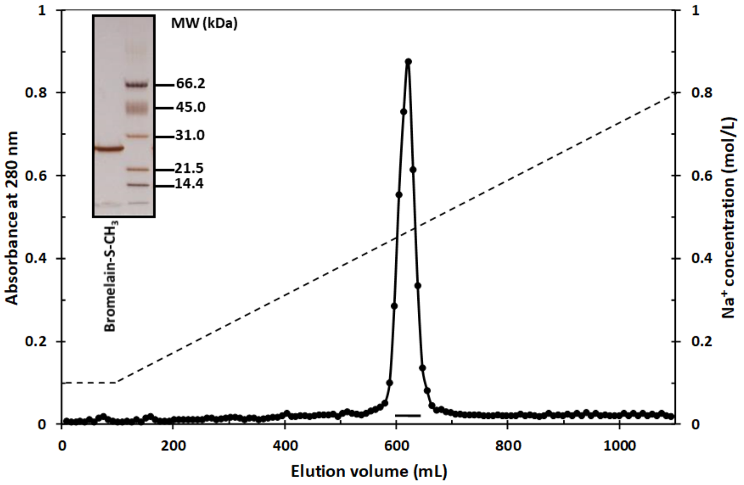

b

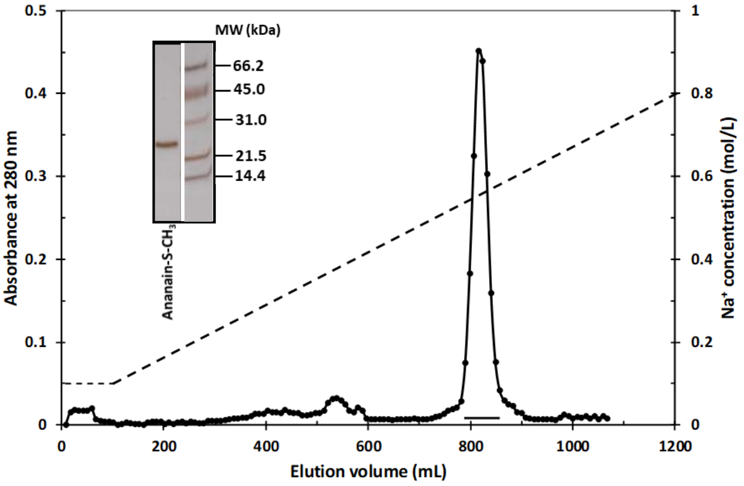

Fractionation of bromelain (**a**) and ananain (**b**) as their S-thiomethylated forms on SP-Sepharose Fast Flow column. Fractions of 8.4 mL were collected at a flow rate of 42.0 mL/h and analyzed by absorbance measurements at 280 nm (filled circles) and Na^+ concentration (dotted line). Fractions were pooled as indicated by solid bars and analyzed by SDS-PAGE. The SDS-PAGE experiments were performed on precast gels (Excel Gel, 245 x 110 x 0.5 mm, gradient 8-18%). Staining was made with silver staining procedures. The uncropped versions of the gels shown as insert in panel (a) and (b) are presented in(**c**) and (**d**) respectively with the corresponding lanes highlighted in red.

c

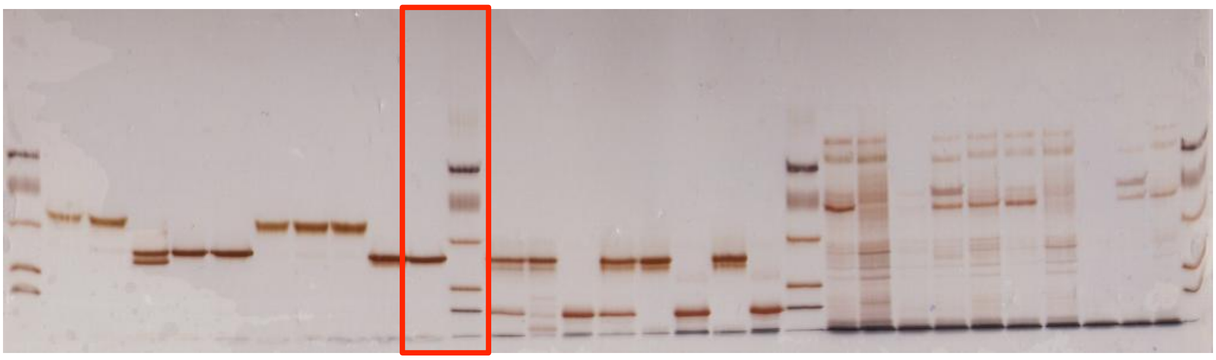

d

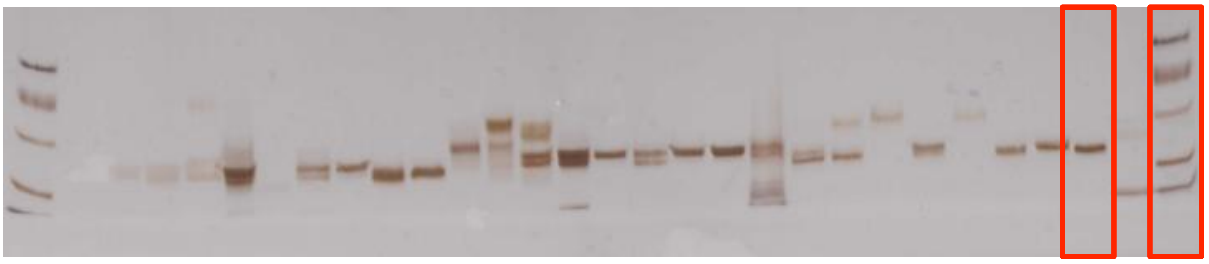

Supplementary Figure 4.

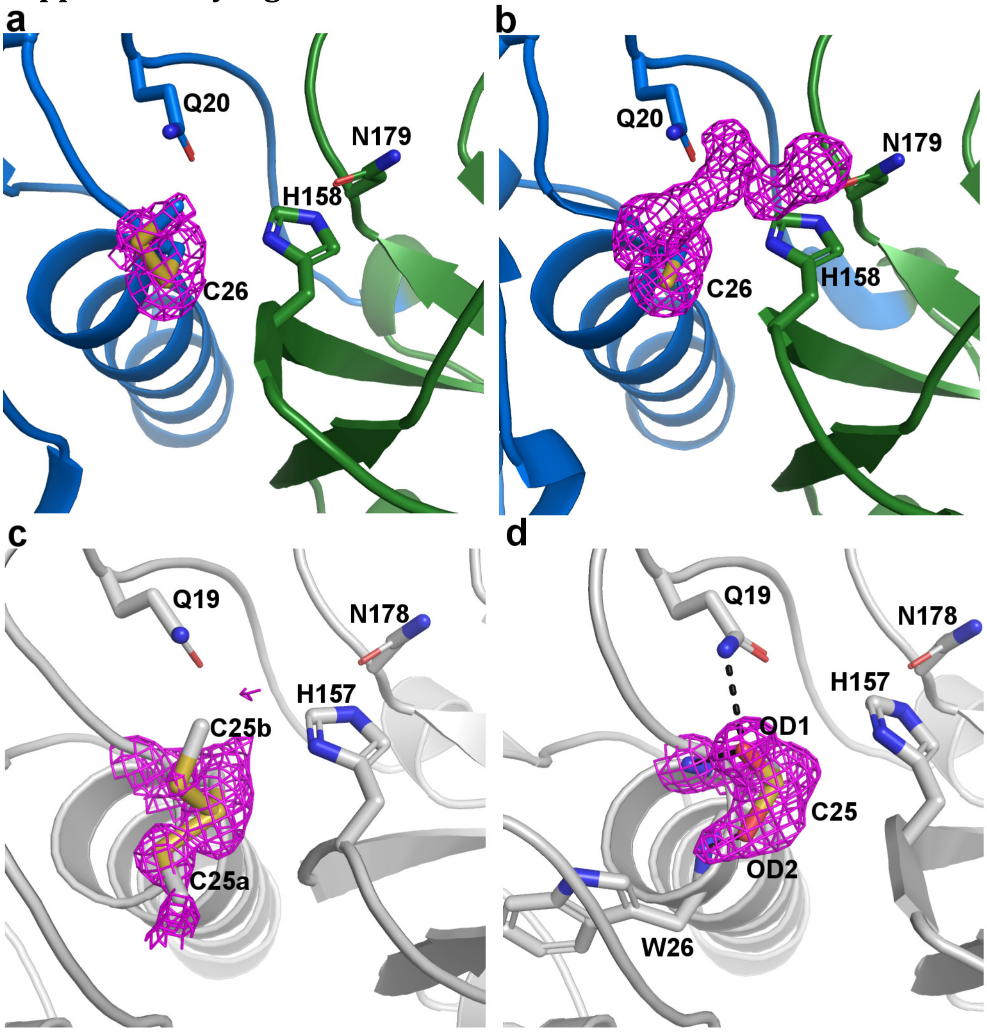

(**a**) Cartoon representation of bromelain (L domain blue and R domain green) with catalytic residues shown as sticks and the 2Fo-Fc electron density map around the thiomethylated Cys26 as magenta mesh at the 1σ level. (**b**) Same as (a) for the monomer A of the bromelain:TLCK complex, the 2Fo-Fc electron density map extending the Cys26 side chain that could not accommodate a TLCK molecule is shown as magenta mesh at the 1σ level. (**c**) and (**d**) same as (a) for the thiomethylated and oxidized forms of ananain respectively (gray). In (c), Lower case letters a and b indicate the two conformations of the thiomethylated C25 residue.

Supplementary Figure 5a.

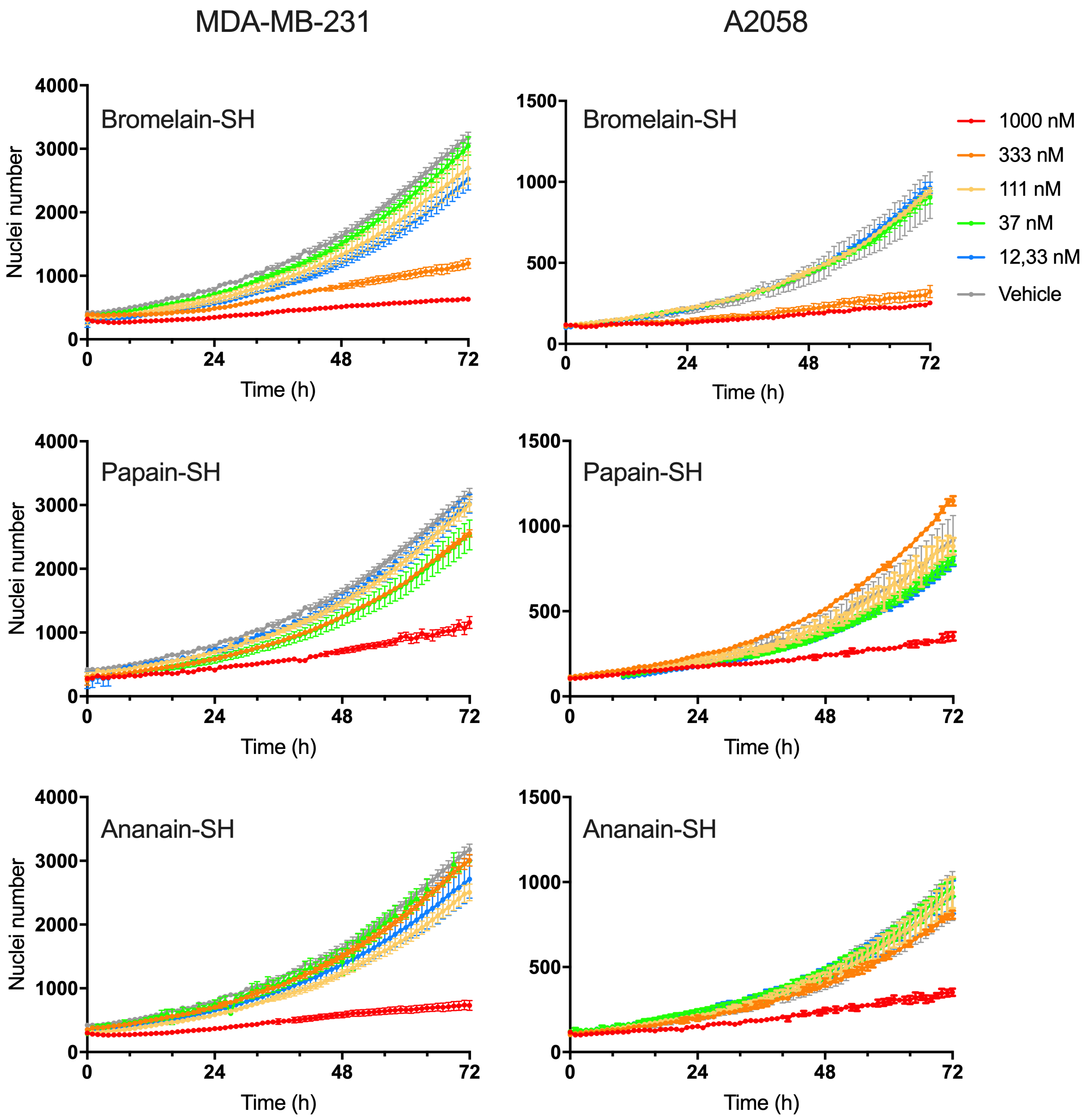

MDA-MB-231 and A2058 cells were treated during 72 h with increasing concentrations of the active (-SH) forms of bromelain, papain and ananain or vehicle only. Live cell imaging was used to measure the number of mKate2+ nuclei. Data are plotted as mean with error bars (SEM, n = 3-4).

Supplementary Figure 5b.

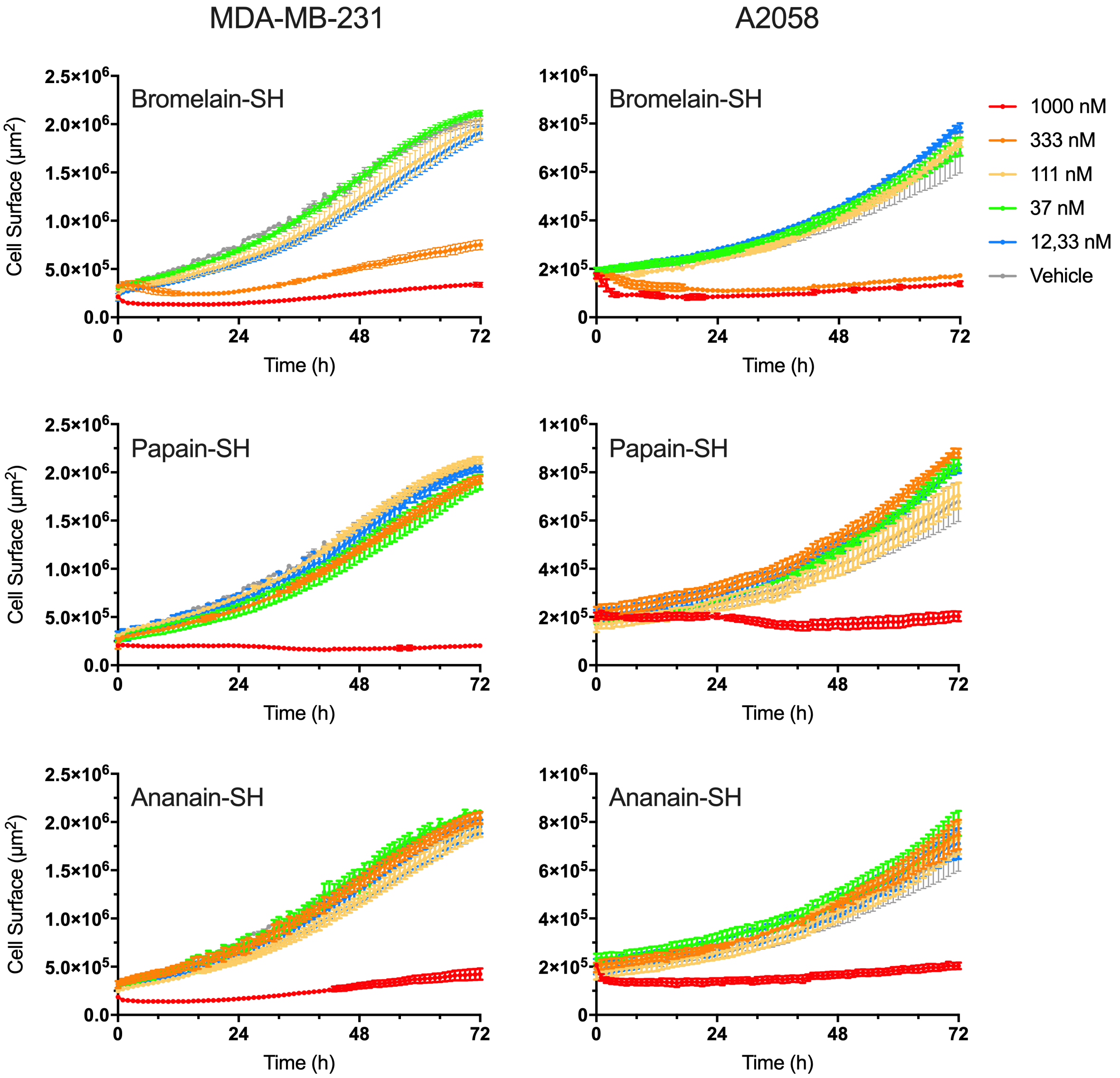

MDA-MB-231 and A2058 cells were treated during 72 h with increasing concentrations of the active (-SH) forms of bromelain, papain and ananain or vehicle only. Live cell imaging was used to measure the cell surface. Data are plotted as mean with error bars (SEM, n = 3-4).

Supplementary Figure 6a.

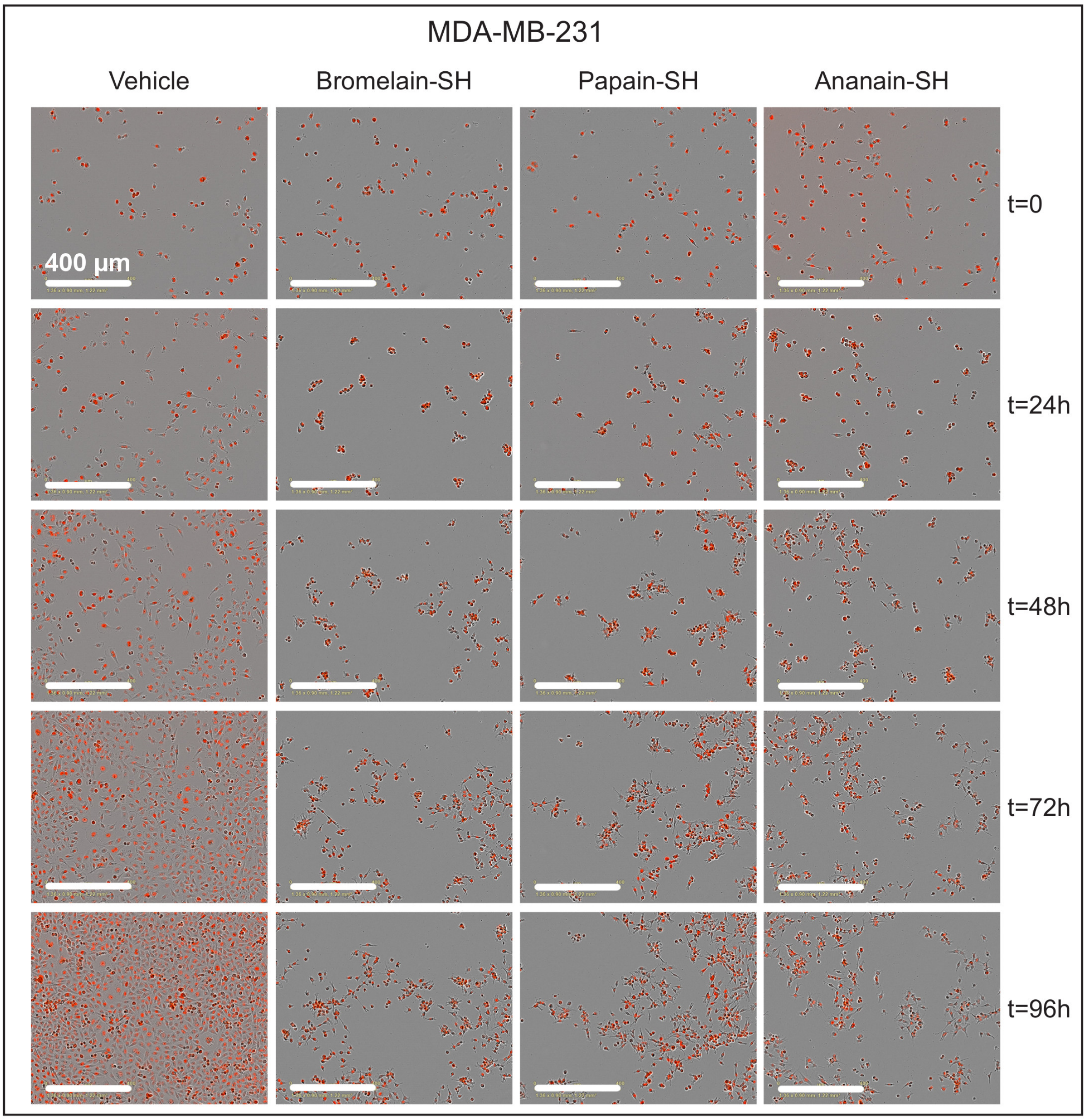

MDA-MB-231 cells were treated with the active (-SH) forms of bromelain, papain and ananain (1 µM) or vehicle only. Live cell imaging was used to obtain pictures of the different culture conditions after 0, 24, 48, 72 and 96 h of treatment. Phase contrast and mKate2 fluorescence images are merged. Scale bar = 400 µm.

Supplementary Figure 6b.

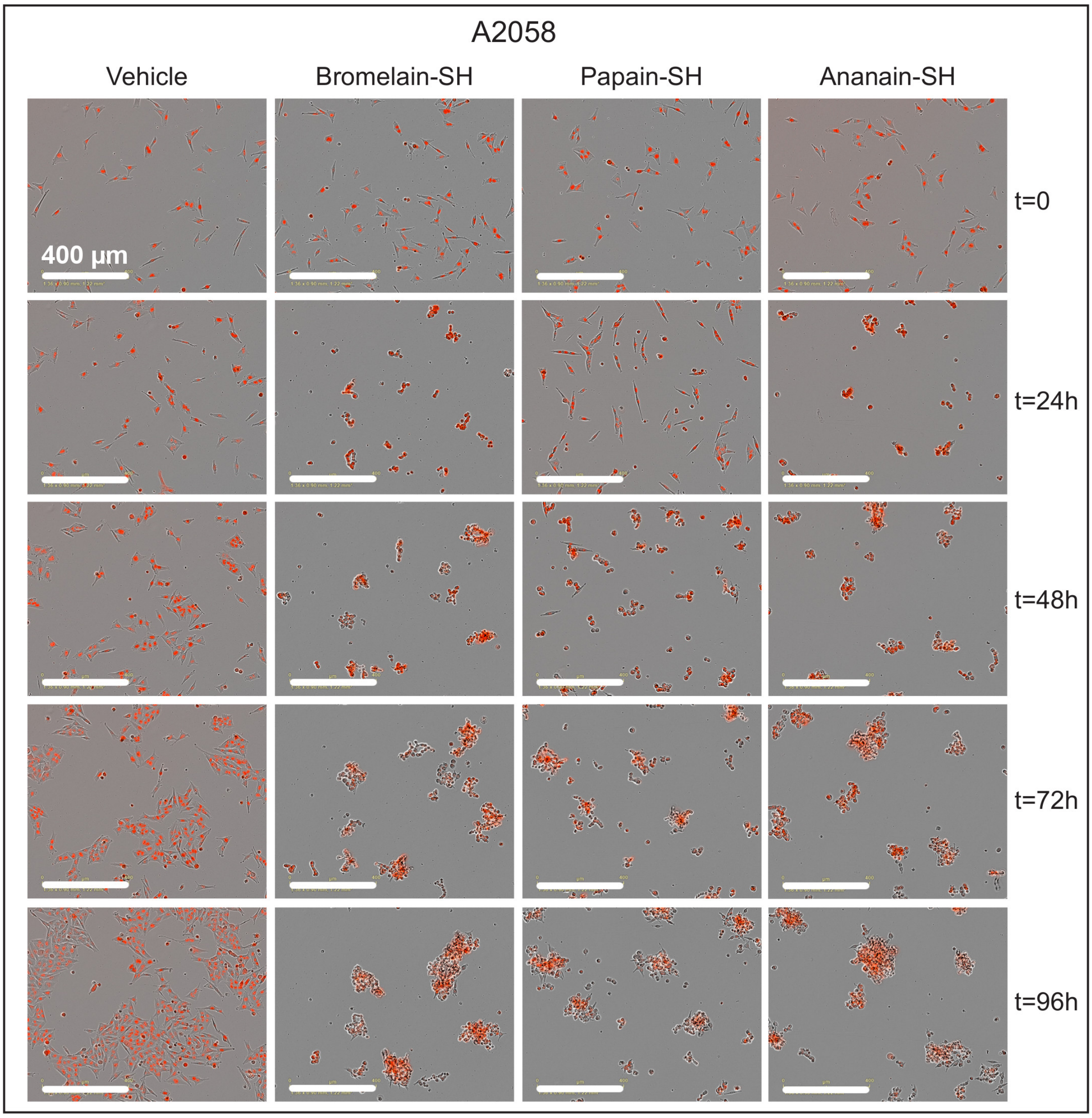

A2058 cells were treated with the active (-SH) forms of bromelain, papain and ananain (1 µM) or vehicle only. Live cell imaging was used to obtain pictures of the different culture conditions after 0, 24, 48, 72 and 96 h of treatment. Phase contrast and mKate2 fluorescence images are merged. Scale bar = 400 µm.

Supplementary Figure 7.

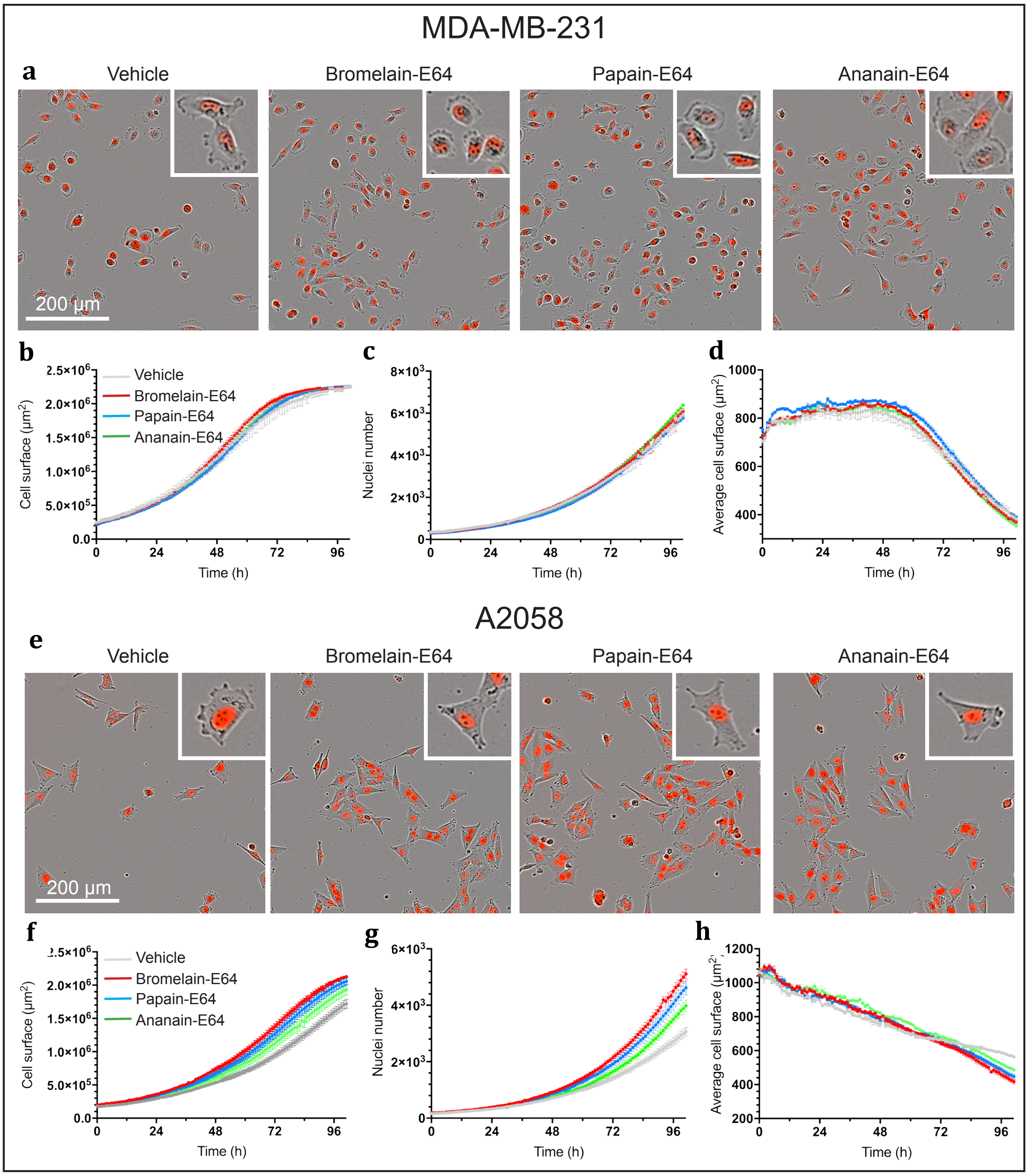

MDA-MB-231 (**a-d**) and A2058 (**e-h**) cells were treated during 100 h with the inactive (-E64) form of bromelain, papain and ananain (1 µM) or vehicle only. Live cell imaging was used to obtain pictures of the different culture conditions (**a** and **e**) and calculate the cell surface (**b** and **f**), the number of mKate2+ nuclei (**c** and **g**) and the average cell surface (**d** and **h**). Pictures were taken after 24h of treatment. Phase contrast and mKate2 fluorescence images are merged. Scale bar = 200 µm. Data are presented as mean ± SEM (n = 3-4). Kolmogorov-Smirnov test did not reveal any statistical differences between the different groups.

Supplementary Figure 8a.

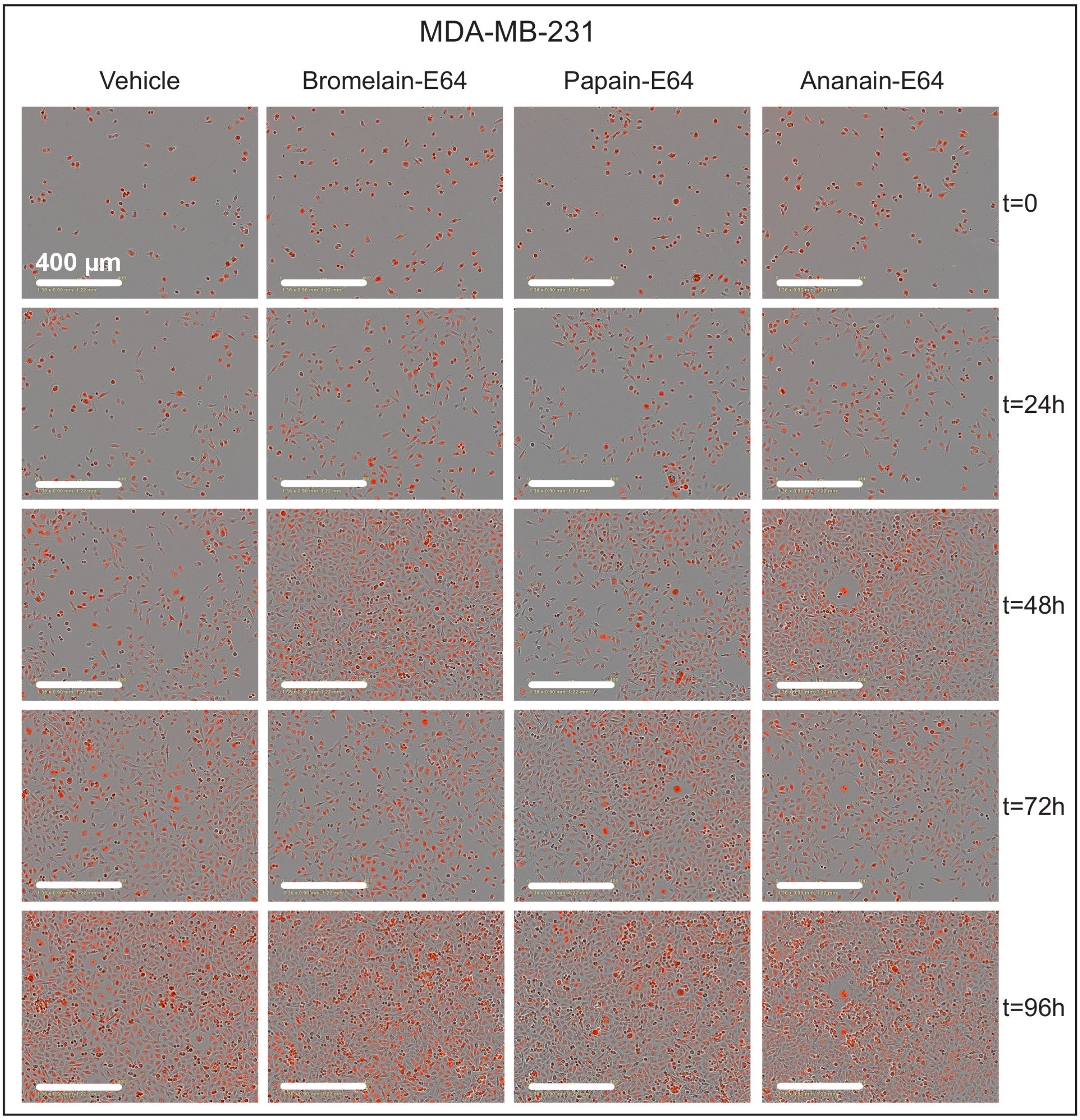

MDA-MB-231 cells were treated with the inactive (-E64) forms of bromelain, papain and ananain (1 µM) or vehicle only. Live cell imaging was used to obtain pictures of the different culture conditions after 0, 24, 48, 72 and 96 h of treatment. Phase contrast and mKate2 fluorescence images are merged. Scale bar = 400 µm.

Supplementary Figure 8b.

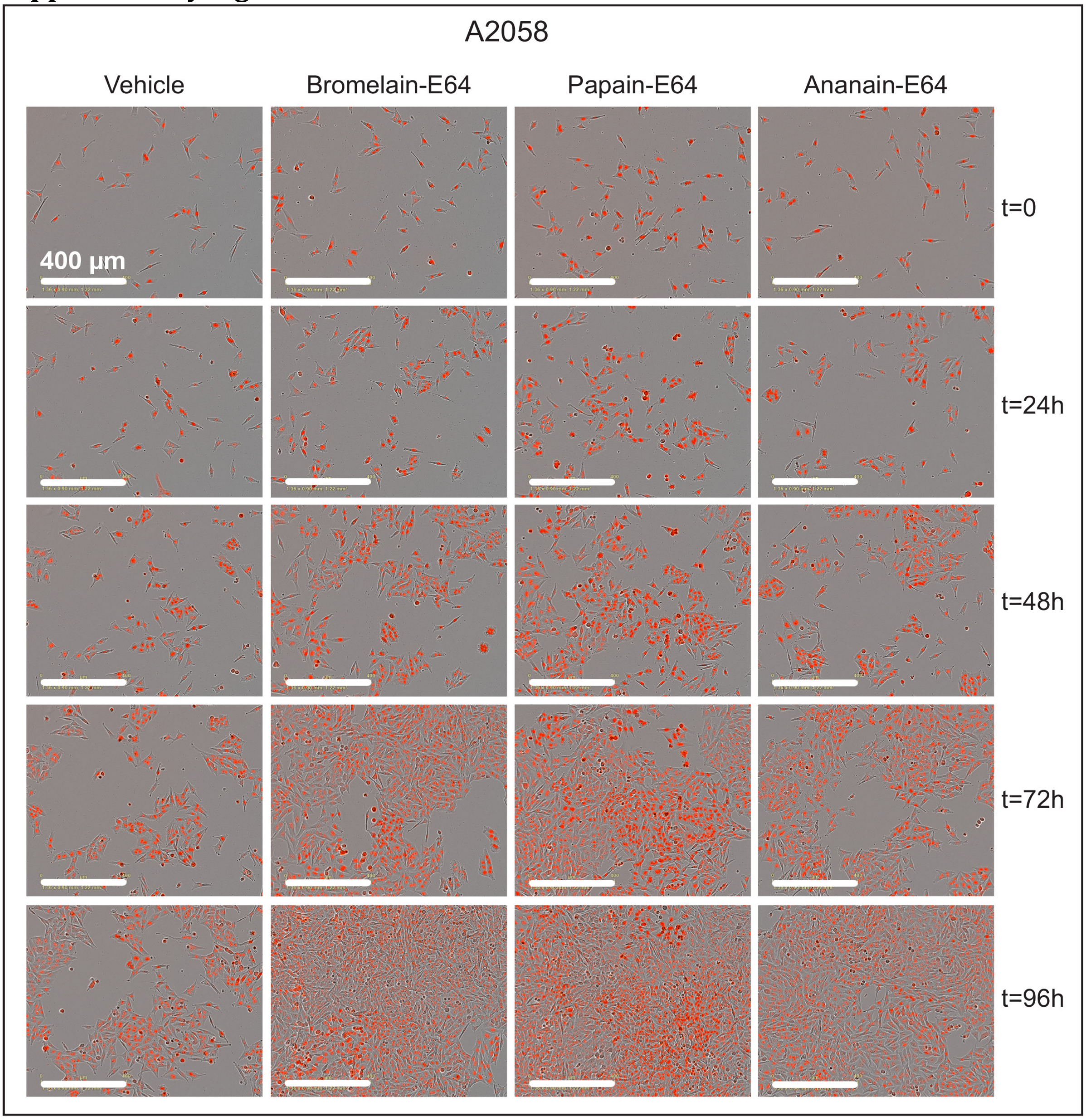

A2058 cells were treated with the inactive (-E64) forms of bromelain, papain and ananain (1 µM) or vehicle only. Live cell imaging was used to obtain pictures of the different culture conditions after 0, 24, 48, 72 and 96 h of treatment. Phase contrast and mKate2 fluorescence images are merged. Scale bar = 400 µm.

Supplementary Figure 9.

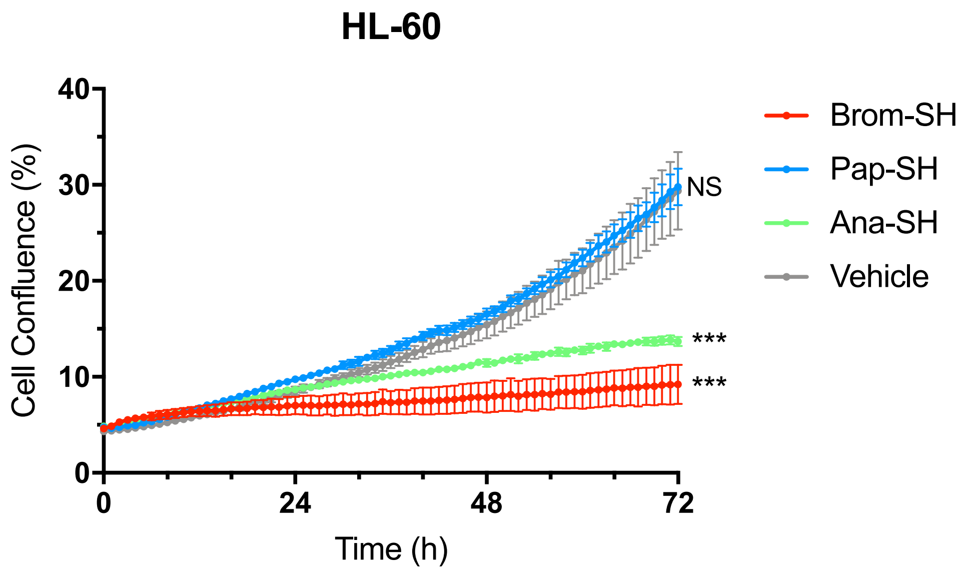

HL-60 cells were treated during 72 h with the active (-SH) forms of bromelain, papain and ananain (1 μM) or vehicle only. Live cell imaging was used to obtain pictures of the different culture conditions and calculate the total cell surface. Data are presented as mean ± SEM (n = 3-4). Kolmogorov-Smirnov test: NS, not significant; ***, $p<0.001$.

Supplementary Figure 10.

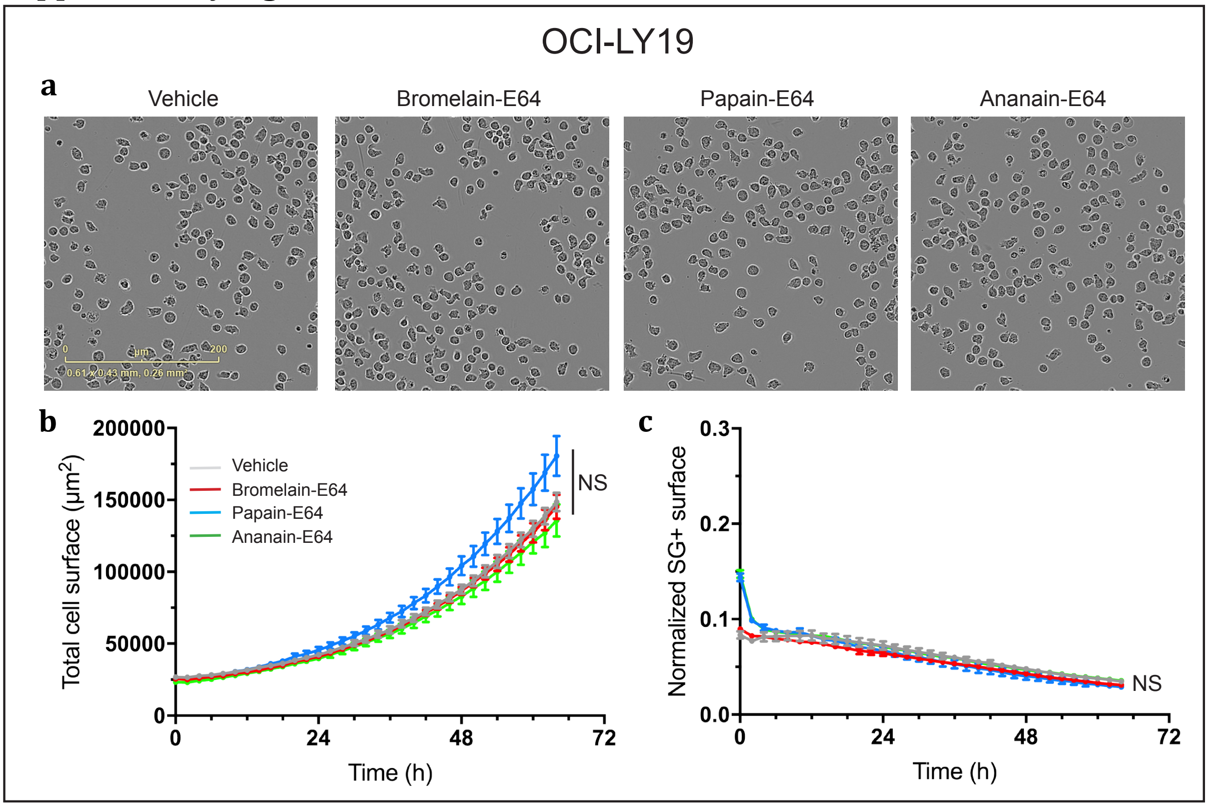

OCI-LY19 cells were treated during 64 h with the inactive (-E64) form of bromelain, papain and ananain (1 μM) or vehicle only. Live cell imaging was used to obtain pictures of the different culture conditions (**a**) and calculate the total cell surface (**b**) and the normalized SYTOX Green (SG) positive cell surface (**c**). Pictures were taken after 64 h of treatment. Scale bar = 200 μm. Data are presented as mean ± SEM (n = 3-4). Kolmogorov-Smirnov test: NS, not significant; ***, p<0.001; ****, p<0.0001.

Supplementary Figure 11.

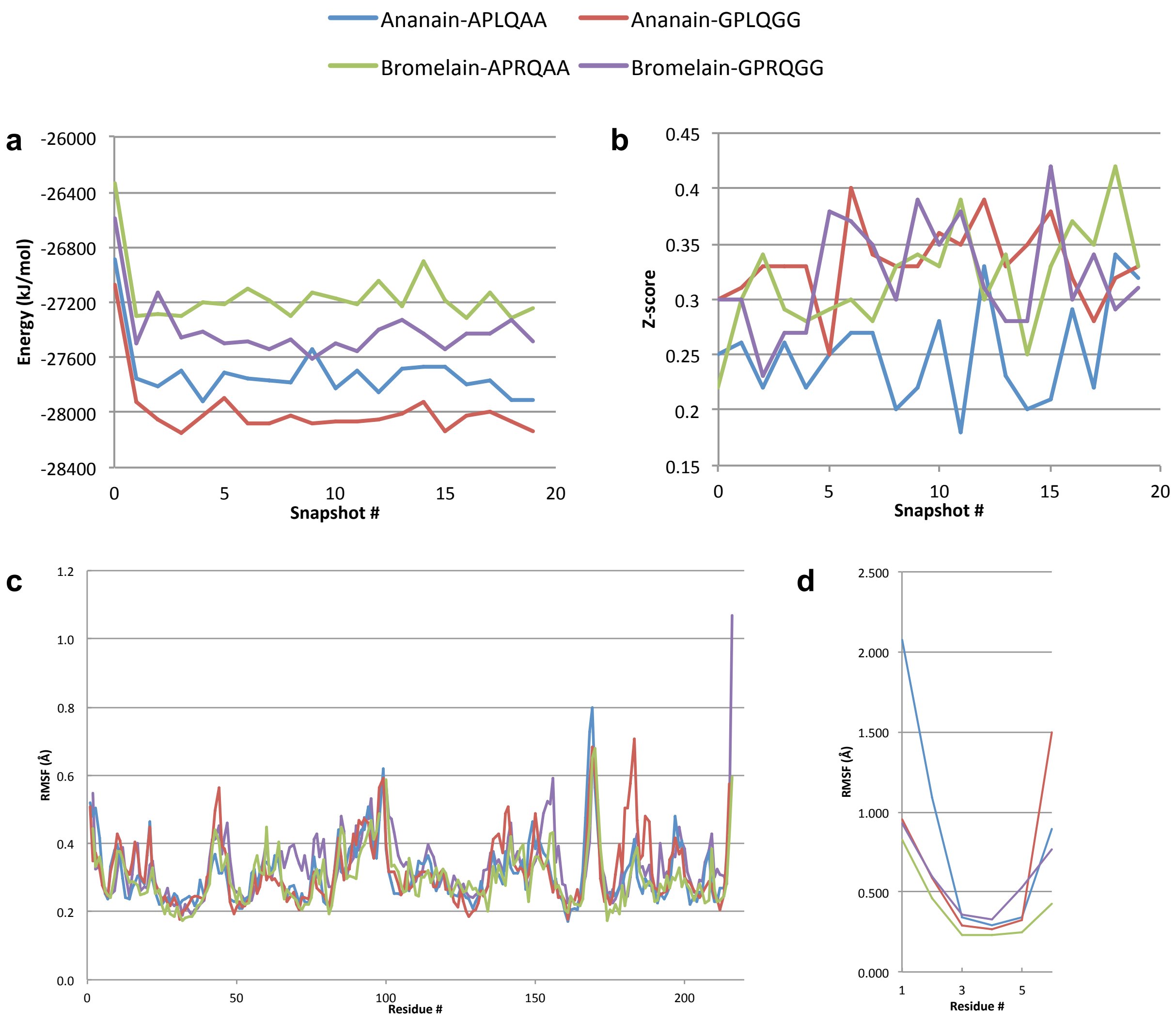

Statistics resulting from the manual modeling of the peptides in the active sites of ananain and bromelain followed by a molecular dynamic refinement procedure. (**a**) and (**b**) respectively present the energy and Z-score of each model obtained after a final energy minimization of the 20 snapshots taken along the molecular dynamic simulations. (**c**) Mean root mean square fluctuation (RMSF) of the main chain atoms of each residue of the protein included in the molecular dynamic refinement. (**d**) Same as (c) for the main chain atoms of the peptide residues.
